# Supplementary material for: Error correction and improved precision of spike timing in converging cortical networks
Source: Cell Rep. 2022 Sep 20;40(12):111383. doi: 10.1016/j.celrep.2022.111383 (PMC9513803; doi:10.1016/j.celrep.2022.111383)
Supplement: Document S1. Figures S1–S7 and Table S1 [file mmc1.pdf]

Cell Reports, Volume *40*

Supplemental information

Error correction and improved precision of spike timing in converging cortical networks

Amir Levi, Lidor Spivak, Hadas E. Sloin, Shirly Someck, and Eran Stark

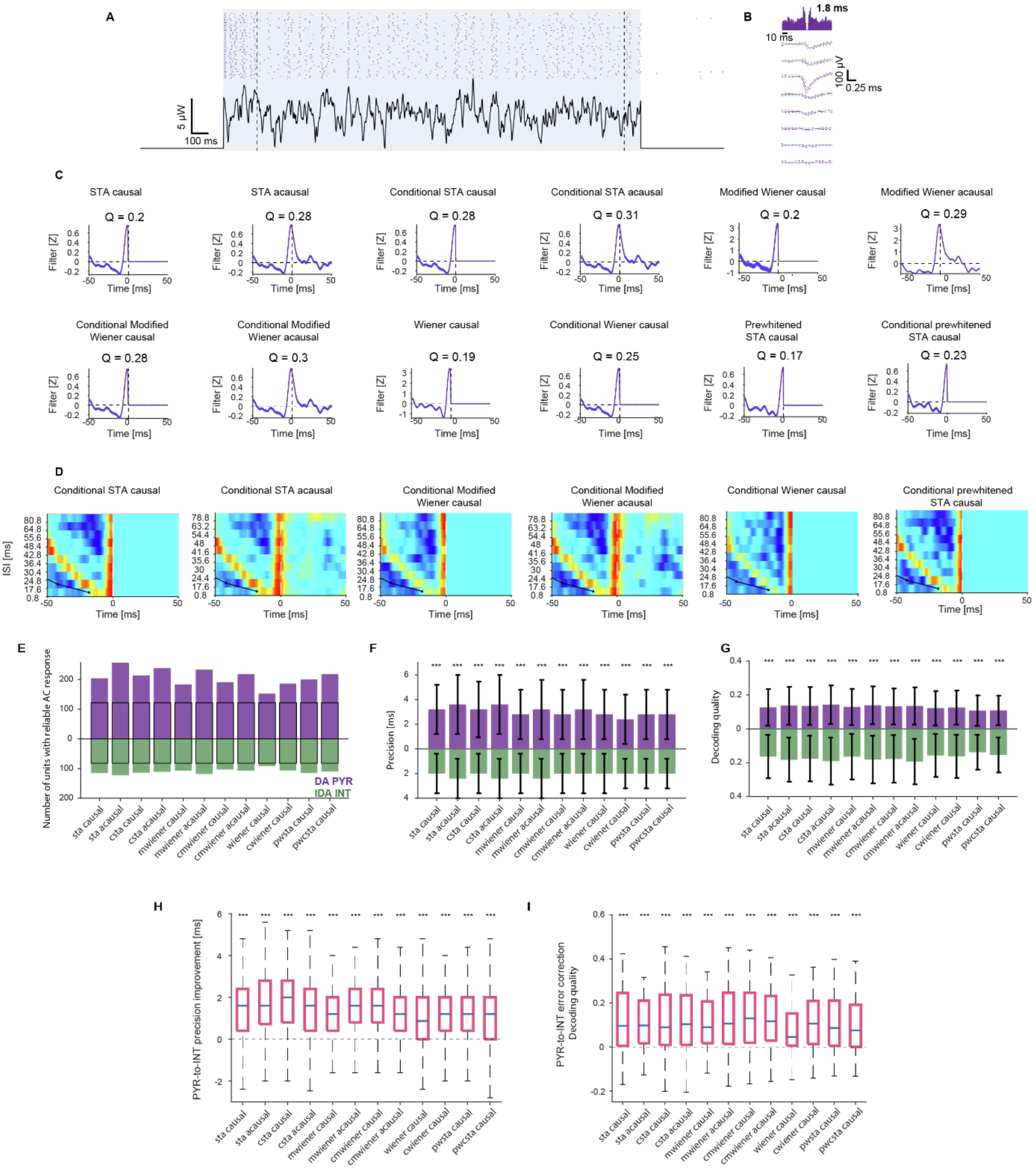

Figure S1. Error correction and improved precision are observed for all twelve filter models employed. Related to Figure 1.

(**A**) Spike trains of an example neocortical DA PYR (same as in **Figure 2C**) during 50 (out of 200) WN trials. (**B**) ACH and waveform of the DA PYR in **A**. (**C**) Twelve models for cross-validated reconstruction. For each model, a filter is generated, based on the WN signal and the spike trains. Decoding quality for all trials is denoted. When conditional models are used, the panel shows the unconditioned filter (not used for reconstruction). (**D**) For conditional models, a filter bank is generated, consisting of one filter for each ISI range. (**E**) The acausal STA filter yields the largest number of AC responsive neocortical DA PYR (257; *purple*) and IDA INT (123; *green*), whereas the causal Wiener filter yields the smallest numbers (152 DA PYR, 591 IDA INT). Black rectangles show the number of AC responsive units common to all filter models (122 DA PYR, 82 IDA INT). (**F**) At the population level, IDA INT exhibit higher precision than DA PYR, regardless of filter model employed. Here and in **G**, */**/***: p<0.05/p<0.01/p<0.001; *U*-test. (**G**) At the population level, IDA INT exhibit higher decoding quality than DA PYR, regardless of filter model. (**H-I**) For connected pairs, precision improvement (**H**) and error correction (**I**) are observed for all filter models. ***: p<0.001, Wilcoxon test.

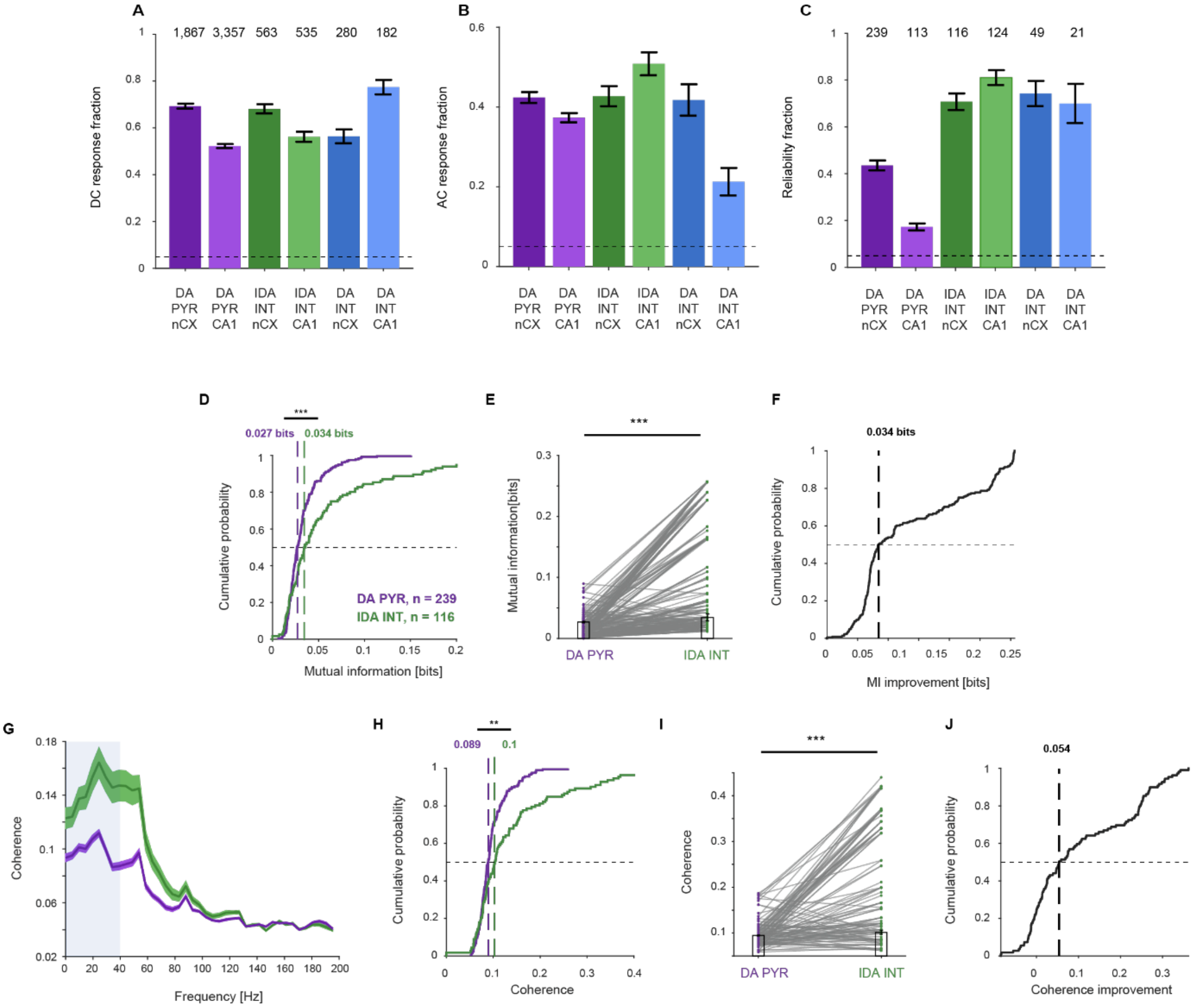

Figure S2. Error correction among the PYR-to-INT interface is evident when quantified using mutual information or spectral coherence. Related to Figure 2.

(**A-C**) Optogenetic WN experiments have low yield. The six categories of units analysed include DA PYR, IDA INT and DA INT, in the neocortex (nCX) and in hippocampal CA1. PYR recorded in PV::ChR2 mice were omitted from the data base. (**A**) Numbers above each bar denote the total number of recorded units of each category. Fraction of units with a "DC response" out of all units recorded during WN experiments. Here and in **B**-**C**, error bars indicate SEM, and horizontal dashed lines indicate chance level (0.05). Of the tested units, 3,998/6,784 (59%) exhibited a DC response. (**B**) Fraction of units with an AC response out of the units with a DC response. Of the units with a DC response, 1,617/3,998 (40%) units exhibited an AC response. (**C**) Fraction of reliable units out of all units with an AC response. Of the units with an AC response, 662/1,617 (41%) units exhibited reliable spike trains. Overall, 662/6,784 (9.8%) of the units recorded during WN experiments exhibited reliable spike trains and were used in this work; the precise numbers for each category are denoted above every bar.

(**D-F**) Mutual information (MI) is higher for IDA INT, compared to DA PYR. (**D**) As a population, IDA INT exhibit higher MI than DA PYR. Here and in **E**, vertical dashed lines indicate median values; **/***: p<0.01/p<0.001, *U*-test. (**E**) In 199/229 (87%) connected pairs, the MI for IDA INT is higher than for presynaptic DA PYR. Here and in **F**, ***: p<0.001, Wilcoxon test. (**F**) MI improvement (median, 0.034 bits) between connected DA PYR/IDA INT pairs.

(**G-J**) Spectral coherence magnitude is higher for IDA INT, compared to DA PYR. (**G**) Averaged coherence profiles of the two populations (mean±SEM). (**H**) As a population, IDA INT exhibit higher coherence (averaged over the 0-40 Hz band; see light blue rectangle in **D**) than DA PYR. (**I**) In 109/143 (76%) connected pairs, the coherence of IDA INT is higher than for presynaptic DA PYR. (**J**) Coherence improvement (median, 0.054) between connected DA PYR/IDA INT pairs.

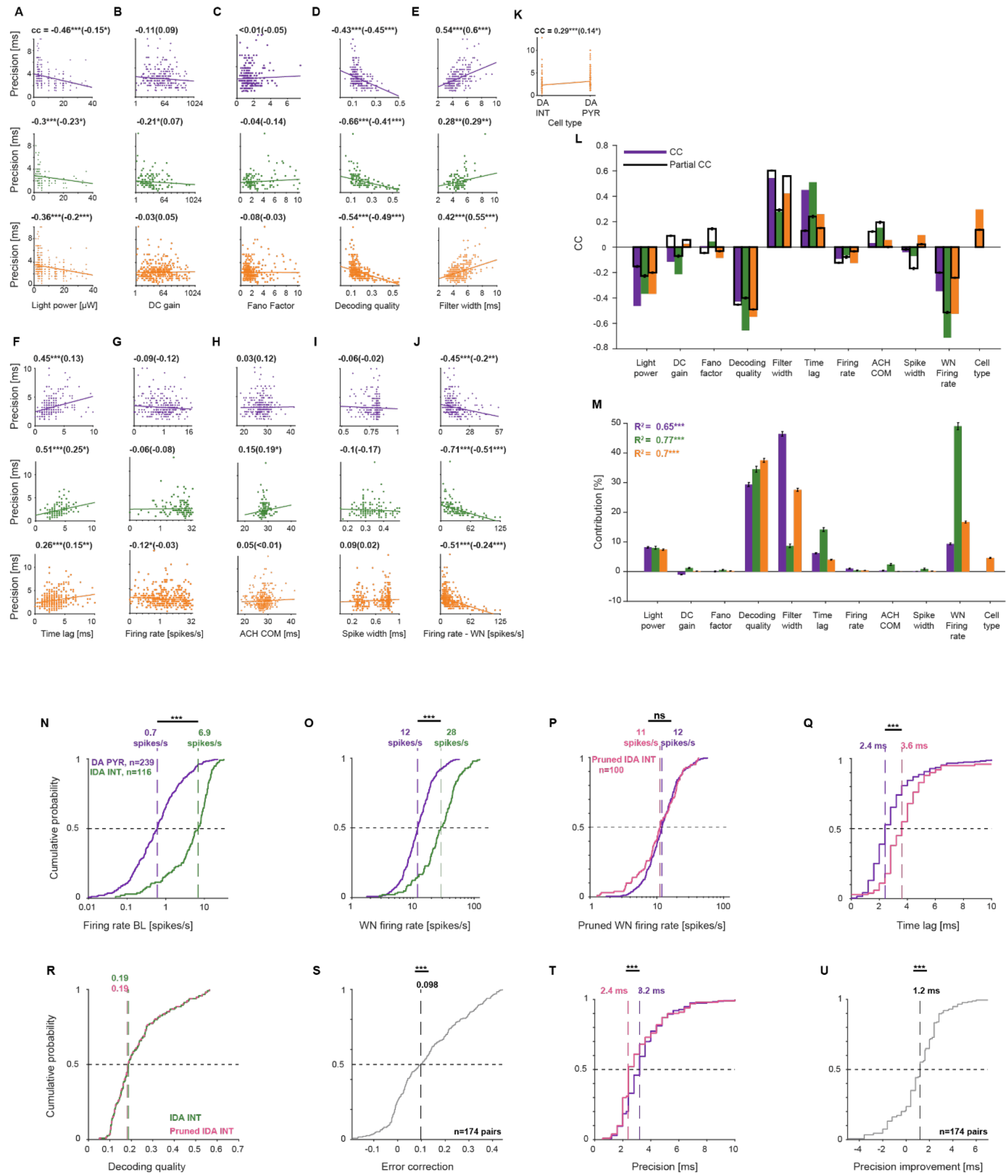

Figure S3. Precision of DA PYR and IDA INT depends mainly on filter width and WN firing rate, but IDA INT precision is preserved when IDA INT WN firing rates are equated to DA PYR WN rates. Related to Figure 2.

(**A-M**) Precision depends mainly o filter width and WN firing rate. (**A**) Scatter plot of the precision and the mean light power of the WN signal (*top*, DA PYR; *middle*, IDA INT; *bottom*, both). Here and in **B-K**, numbers indicate rank correlation coefficients (cc); numbers in parentheses indicate partial cc; */**/***: $p<0.05/p<0.01/p<0.001$, permutation test. **B-K**, Same as **A**, for other parameters. (**B**) DC gain is the mean firing rate during WN trials, divided by the mean spontaneous firing rate in the lack of light. (**C**) Fano factor is the variance/mean spike count over different WN trials. (**D**) Decoding quality, Q. (**E**) Filter width is the full width at half maximum (FWHM) of the filter. (**F**) Time lag. (**G**) Firing rate is the mean spontaneous firing rate. (**H**) ACH COM is the center of mass of the ACH. (**I**) Spike width is the trough-to-peak duration of the spike waveform. Firing rate, ACH COM, and spike width (**G-I**) are computed in the lack of light. (**J**) WN firing rate is the mean firing rate during WN trials. (**K**) Cell type is a binary indicator that equals 1 for DA PYR and 0 for IDA INT. (**L**) Summary of the cc's and partial cc's between precision and every tested parameter. Here and in **M,** error bars

indicate SEM. Filter width and time lag are positively correlated with precision, whereas decoding quality and WN firing rate are negatively correlated. Thus, precision is higher (lower values) when decoding quality is high (higher values), when the WN firing rate is high, when the filter is narrower, and when time lag is short. (**M**) The contribution of each parameter (the product of the rank regression coefficient and the cc; can be negative for some parameters) to the total variability of the precision explained by the model (R^2). Within the IDA INT population, the major source of contribution is from the WN firing rate. In contrast, the major source of contribution for DA PYR is filter width. For the combined population, the cell type parameter makes a minor contribution. Thus, WN firing rate is important in generating more precise IDA INT, but is not a major contributor for the difference in precision between the two populations.

(**N-U**) Error correction and improved precision are preserved when IDA INT firing rate during WN trials is equated to DA PYR rates. (**N**) CDFs of the baseline (BL) firing rate (the mean spontaneous firing rate, in the lack of light) of the DA PYR and IDA INT. Here and in **O-U**, vertical dashed lines show group medians; ns/***: p>0.05/<0.001, *U*-test. During baseline, IDA INT firing rates are an order of magnitude higher than DA PYR rates (medians: 6.9 vs. 0.6 spikes/s). (**O**) CDFs of the WN firing rates. Median IDA INT firing rates are approximately double the DA PYR firing rates (medians: 28 vs. 12 spikes/s; p<0.001, *U*-test). (**P**) For each IDA INT, WN firing rate was pruned by removing the spikes least likely to have emanated from the WN input. After pruning, the median firing rates of the IDA INT population are not higher than the firing rates of the DA PYR (DA PYR: 12 spikes/s; IDA INT: 11 spikes/s, pink dashed line). (**Q**) CDFs of the time lags of DA PYR and IDA INT after pruning. Akin to the IDA INT, pruned IDA INT exhibit longer time lags compared to the DA PYR (medians: 3.6 vs 2.4 ms; see **Figure 2F**). (**R**) Pruning does not change IDA INT decoding quality (medians: 0.19 vs. 0.19). (**S**) Distribution of the decoding quality between pairs of connected DA PYR and pruned IDA INT (see **Figure 2J**). The decoding quality of the pruned IDA INT is 0.098 higher than the decoding quality of the presynaptic DA PYRs (here and in **H**,***: p<0.001, Wilcoxon test), indicating error correction. (**T**) CDFs of the precision of the DA PYR and of the IDA INT after pruning. The pruned IDA INT exhibit high precision despite reduced firing rates. In particular, pruned IDA INT are more precise than DA PYR (median: 2.4 vs 3.2 ms; p<0.001, *U*-test; see **Figure 2H**). (**U**) Distribution of the precision difference between pairs of connected DA PYR and pruned IDA INT (see **Figure 2L**). The precision of the pruned IDA INT is 1.2 ms higher than the precision of the presynaptic DA PYRs.

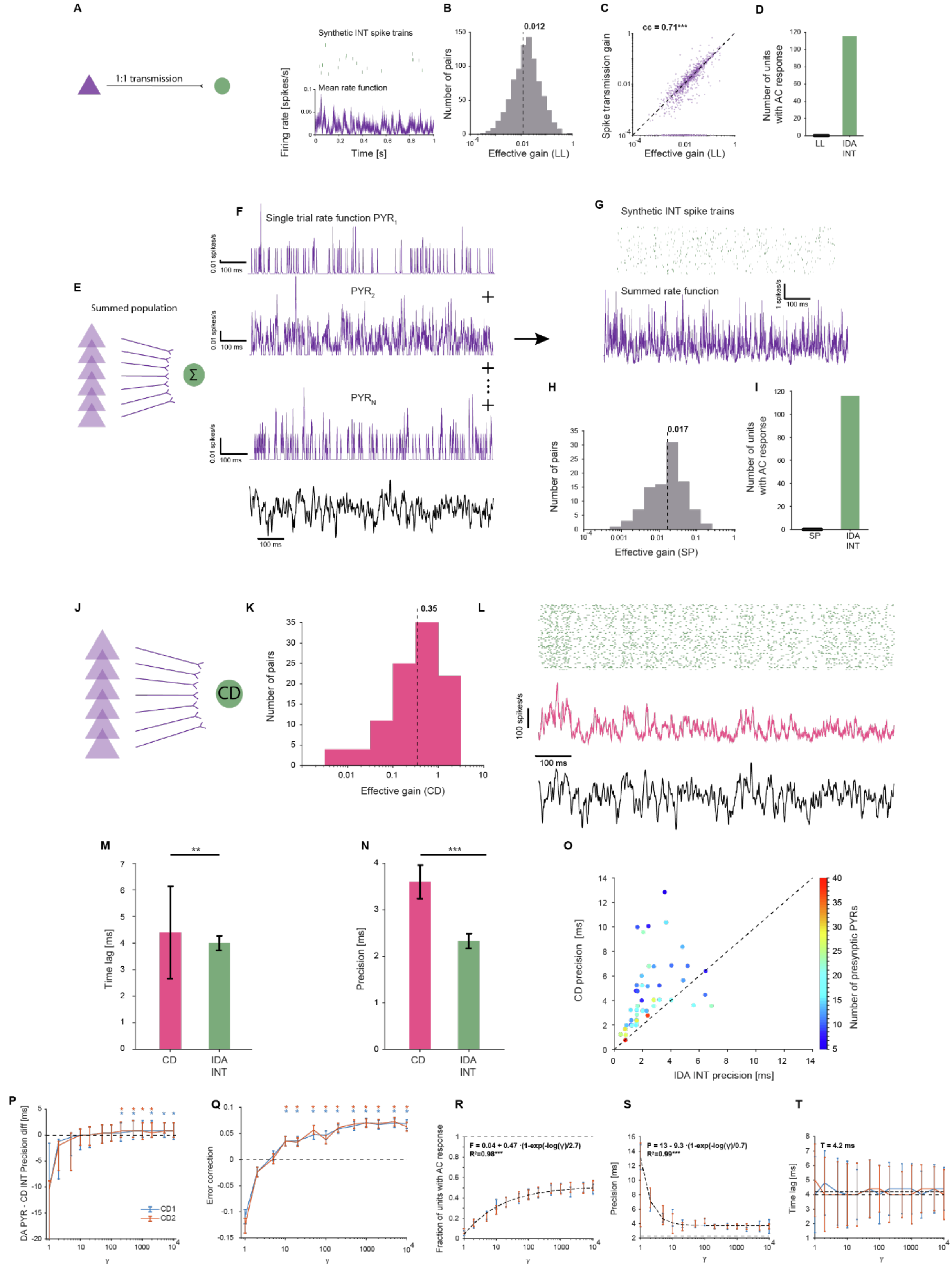

Figure S4. Coincidence detection transmission models produce error correction and temporally precise spiking in postsynaptic IDA INT. Related to Figure 4.

(**A-D**) Labelled line (LL) transmission model does not generate spiking in postsynaptic sINT. (**A**) *Left*, Illustration of a LL transmission model, showing a presynaptic neocortical DA PYR (purple triangle) and a postsynaptic IDA INT. *Right, bottom*, Mean sINT rate function, averaged over all trials (n=200) of the example DA PYR spike trains. *Right, top*, The resulting sINT spike trains during a subset of 50 WN trials. The sINT does not exhibit an AC response to the WN. (**B**) In the presence of a fixed input (the WN), the *effective* transmission

gain can be estimated by n_1/n_0, where n_0 (or n_1) refers to the number of presynaptic PYR spikes (or the number of sINT spikes produced, excluding background spikes) during WN. For the LL sINT spike trains, the effective gain is smaller than the spike transmission gain (median [IQR]: 0.012 [0.002 0.029]; p<0.001, Wilcoxon test; see **Figure 4C**). (**C**) Spike transmission gain and effective transmission gain are correlated (cc, 0.71; ***: p<0.001, permutation test). (**D**) Using the LL approach, none of the sINT exhibit an AC response.

(**E-I**) Summed population (SP) transmission model does not generate spiking in postsynaptic sINT. (**E**) Illustration of SP transmission model for an sINT (green), generated by linear summation of all inputs from the presynaptic DA PYR (purple triangles) to the modeled sINT. (**F**) For each presynaptic PYR, a rate function was generated. (**G**) For every trial, all rate functions were added together, producing a summed rate function (shown averaged over all trials; *bottom*). On every trial, the summed rate function was used to stochastically generate an sINT spike train. In the example, N=16 simultaneously-recorded presynaptic PYR were used. sINT spike trains during 50 trials are shown (*top*). (**H**) The effective gain between the summed presynaptic PYR and their postsynaptic sINT (median [IQR]: 0.017 [0.005 0.029], 109 pairs) is higher than the effective gain for the LL model (p=0.03, *U*-test). (**I**) Using the SP with added background activity, none of the sINT exhibit an AC response.

(**J-T**) coincidence detection (CD) transmission models generate precise spiking in postsynaptic sINT. (**J**) In the CD transmission models, inputs that arrive simultaneously are combined in a supra-linear manner. (**K**) The effective gain between the presynaptic PYR and their postsynaptic CD sINT (median [IQR]: 0.35 [0.10 0.78], 109 pairs) is higher than the effective gain for the SP model (p<0.001, Wilcoxon test). (**L**) Example of a CD generated rate function (*middle*, pink line) and the resulting sINT spike trains (*top*). (**M**) The time lag is longer for CD sINT, compared to IDA INT (medians: 4.4 vs. 4 ms; **: p<0.01, Wilcoxon test). (**N**) Precision of the CD sINT is poorer than that of the IDA INT (medians: 3.6 vs. 2.3 ms; ***: p<0.001, Wilcoxon test). (**O**) CD sINT and IDA INT precision are particularly similar when the number of simultaneously-recorded presynaptic PYR is large. (**P**) Two CD models were employed: CD1 (multiplicative nonlinearity) and CD2 (exponential summation). For both models, precision difference between pairs of connected DA PYR and the corresponding postsynaptic CD sINT was evaluated as a function of γ, a scalar non-negative non-linearity factor. The median difference is larger than zero (i.e., the CD model exhibits precision improvement) for $\gamma>20$. Precision improvement becomes consistent for $\gamma\geq200$ for both models (*: p<0.05, Wilcoxon test). Here and in **Q-T**, error bars indicate SEM over sINT. (**Q**) The median error correction is larger than zero for $\gamma>5$. Error correction becomes consistent for $\gamma\geq10$ for both models (*: p<0.001, Wilcoxon test). (**R**) For both models, the fraction of CD sINT that exhibit an AC response as a function of γ is monotonically increasing and achieves 95% of the asymptotic value (0.51) at $\gamma=127$ (51% of units exhibit an AC response; 95% confidence interval: [43% 0.51%]; fit to an exponential model: $R^2=0.99$, p<0.001, F-test). Here and in **S, T** the fit was for CD1. In **M-O**, CD1 was used with $\gamma=1000$. (**S**) The median CD sINT temporal precision improves as a function of γ, and reaches 95% of the asymptotic value (P=3.75 ms) at $\gamma=9$ (95% confidence interval, [3.56 3.94] ms; $R^2=0.98$, p<0.001). (**T**) The median CD sINT time lag is fixed, regardless of γ (constant fit to the data: T=4.2 ms).

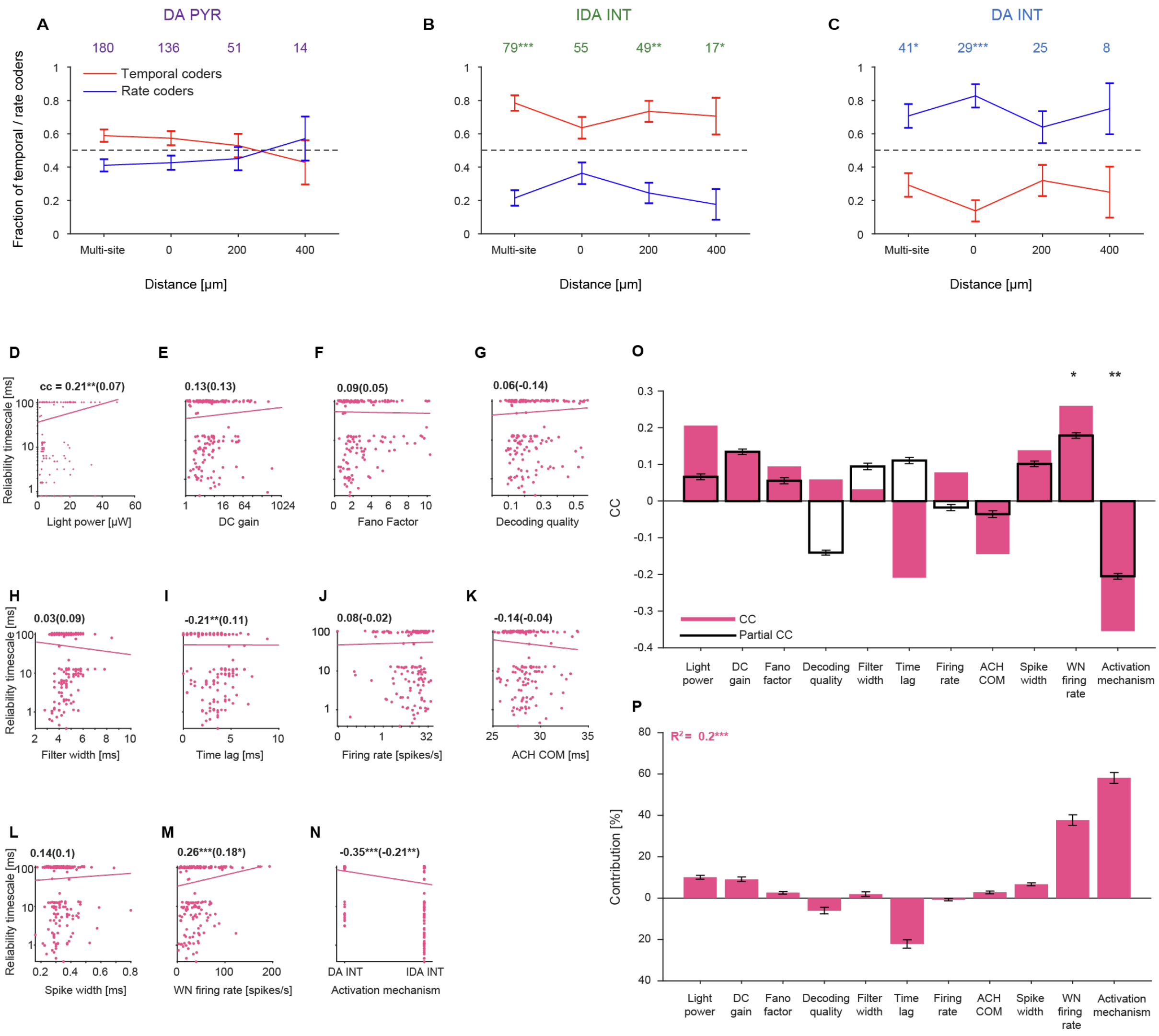

Figure S5. Reliability timescale of INT depends mainly on the activation mechanism. Related to Figure 5. (**A-C**) Reliability timescale of INT is independent of the distance from the illuminated shank. Fraction of rate (blue) and temporal coders (red) as a function of distance from the illuminated shank. Most IDA INT/DA INT are temporal/rate coders, respectively; DA PYR exhibit mixed coding behavior. */**/***: p<0.05/p<0.01/p<0.001, *G*-test.

(**D-P**) Reliability timescale of INT depends on WN firing rate and the activation mechanism. (**D**) Scatter plot of the reliability timescale and mean light power of the WN signal for a combined population of DA and IDA INT. (**E-M**), Same as **D**, for the same parameters used in **Figure S3B-J**. (**N**) Activation mechanism is a binary indicator that equals 0 for direct activation and 1 for indirect activation. (**O**) cc's and partial cc's between reliability timescale and every tested parameter. Here and in **P,** error bars indicate SEM. WN firing rate is positively correlated with reliability timescale, whereas activation mechanism is negatively correlated. Thus, reliability timescale is lower (tending towards temporal coding) when the WN firing rate is lower, and when the unit is indirectly activated. (**P**) The contribution of each parameter to the total variability of reliability timescale explained by the model (R^2). For the combined population, the activation mechanism makes the highest contribution.

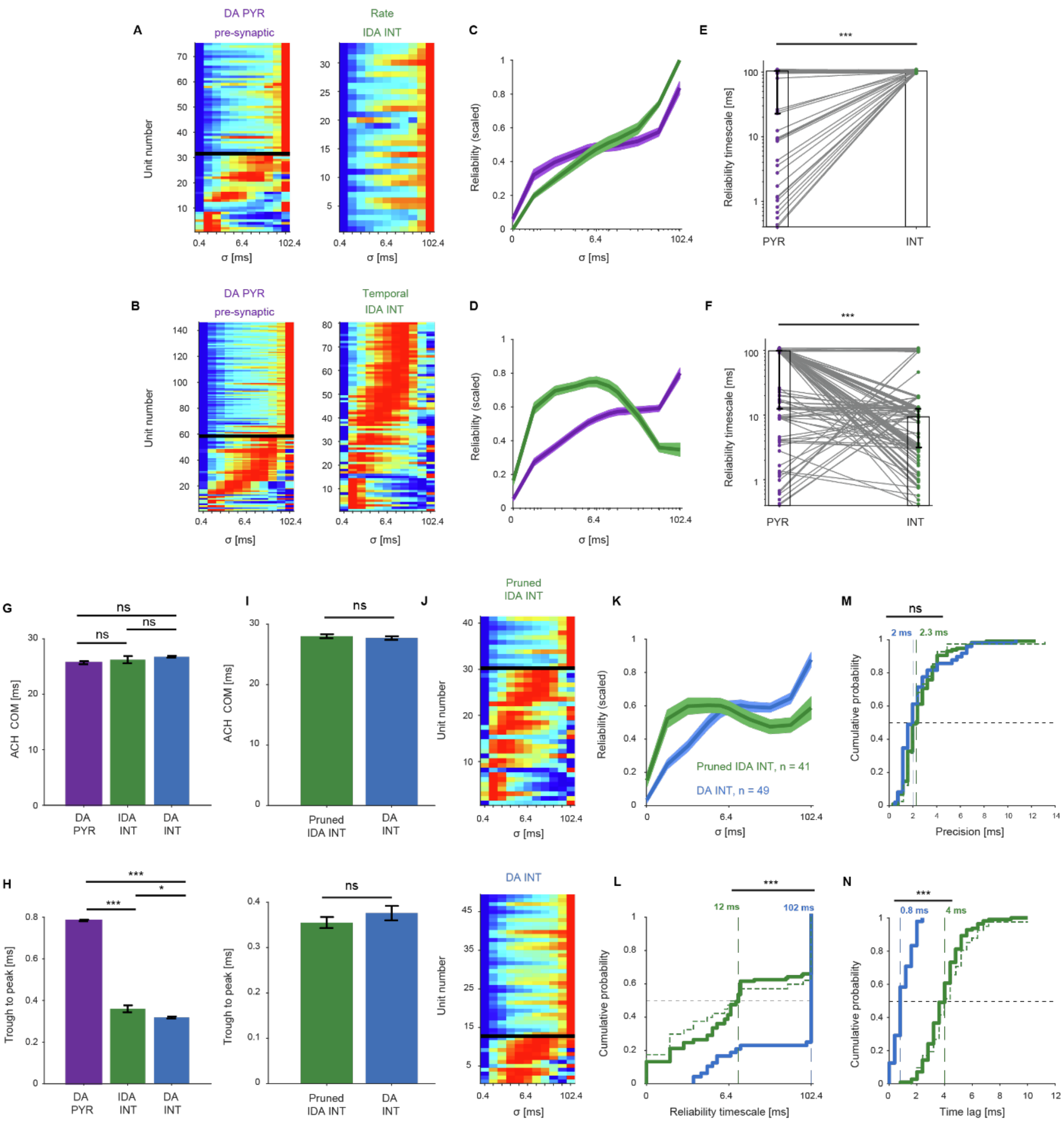

Figure S6. IDA INT reliability timescale is not inherited from DA PYR, and IDA INT are high precision temporal coders. Related to Figure 5.

(**A-F**) IDA INT reliability timescale is not inherited from DA PYR. (**A**) Stacked reliability profiles (scaled to the 0-1 range and sorted according to peak) of neocortical DA PYR (n=75; *left*) presynaptic to IDA INT classified as rate coders (n=33; *right*). Reliability profiles are arranged with temporal/rate coders below/above the black lines. (**B**) Same as **A**, for DA PYR (n=146; *left*) presynaptic to IDA INT classified as temporal coders (n=80; *right*). (**C**) The reliability profiles of the two populations, averaged (mean±SEM) over all "rate IDA INT" and their presynaptic DA PYR. At the group level, DA PYR presynaptic to "rate IDA INT" act as rate coders. (**D**) Same as **C**, for temporal IDA INT. At the group level, the DA PYR presynaptic to "temporal IDA INT" also act as rate coders. (**E**) The reliability timescale of each rate IDA INT is plotted, along with the reliability timescale of all corresponding presynaptic PYRs. Grey lines indicate inferred monosynaptic connectivity. In 54/75 (72%) of the pairs, the reliability timescales are the same. In all other cases, the reliability timescale of the presynaptic PYRs is lower than that of their IDA INT peers. Thus, the null hypothesis of similar reliability timescales for the pre- and postsynaptic units is rejected for this population (***: $p<0.001$, Wilcoxon test). (**F**) Same as **E**, for temporal IDA INT. In 113/146 (77%) of the pairs, the reliability timescale is lower for the IDA INT than for the presynaptic DA PYR (***: $p<0.001$, Wilcoxon test).

(**G-N**) Neocortical IDA INT with timing and waveform properties of optically-verified PV cells are high precision temporal coders. (**G-H**) DA INT and IDA INT have similar second-order spontaneous spike timing statistics (quantified here using the ACH center of mass, *left*) but may differ in their spike waveforms (quantified

using the trough-to-peak duration, *right*). IDA INT and DA INT are distinct from DA PYR. ns/*/***: p>0.05/p<0.05/0.001, *U*-test. (**I**) The neocortical IDA INT population (n=116) was pruned such that the remaining IDA INT ("pruned IDA INT"; n=41) and all DA INT (n=49) had similar spike timing (*top*) and spike waveforms (*bottom*). (**J**) Stacked reliability profiles of pruned IDA INT (*top*) and DA INT (*bottom*) populations, scaled and sorted according to the timescale of the peak. (**K**) Same as **C** for the two population. At the group level, DA INT act as rate coders, whereas pruned IDA INT act as temporal coders. (**L**) CDFs of the global maxima of the reliability profiles of the two populations. Here and in **M** and **N**, vertical dashed lines show group medians; ns/***: p>0.05/p<0.001, *U*-test. The pruned IDA INT exhibit reliability timescales consistent with temporal coding, as does the complete population of IDA INT (green dashed line). (**M**) DA INT and pruned IDA INT exhibit similar precision. (**N**) CDFs of the time lags of the two populations. The median time lag for DA INT is 3.2 ms shorter than for pruned IDA INT.

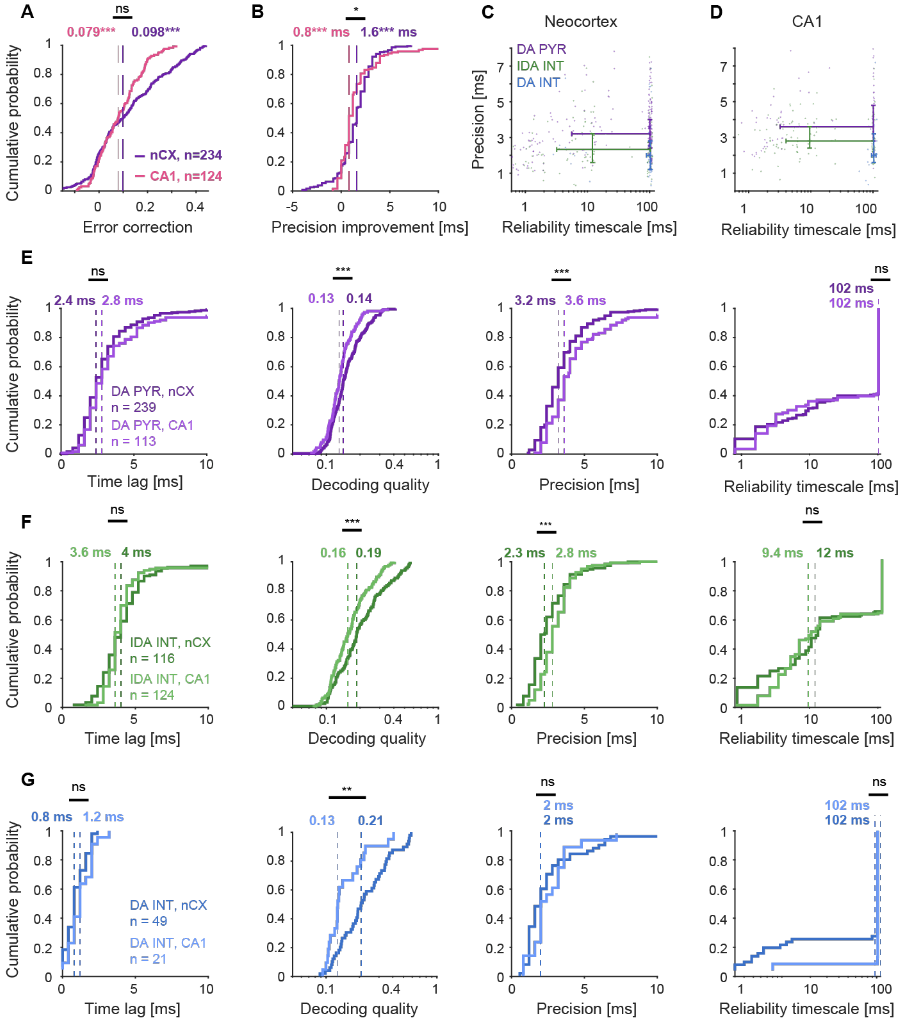

Figure S7. Precision improvement is larger in neocortex than in CA1. Related to Figure 7.

(**A**) Error correction between pairs of connected DA PYR and IDA INT is evident in neocortex (purple) and in CA1 (pink). Error correction is not consistently different between neocortex and CA1. Here and in **E**, ***: p<0.001, Wilcoxon test; ns/*: p>0.05/p<0.05, *U*-test.

(**B**) Precision improvement between connected DA PYR and IDA INT is higher in the neocortex than in CA1.

(**C-D**) Summary of the relations between precision and reliability timescale for all neocortical and CA1 units. Error bars represent population medians and IQR. DA PYR resemble low precision rate coders (cf. **Figure 1G**); IDA INT resemble high precision temporal coders (**Figure 1H**); and DA INT resemble high precision rate coders (**Figure 1I**). In both regions, INT exhibit higher precision than PYR, and DA INT act as rate coders. IDA INT act as temporal coders and exhibit higher precision than DA PYR.

(**E**) Neocortical DA PYR exhibit higher decoding quality and precision than DA PYR in CA1 (*center*) while having similar time lags (*left*) and reliability timescales (*right*). Here and in **F-G**, ns/**/***: p>0.05/p<0.01/p<0.001, *U*-test).

(**F**) Neocortical IDA INT exhibit higher decoding quality and precision than IDA INT in CA1, while exhibiting similar time lags and reliability timescales.

(**G**) Neocortical DA INT exhibit higher decoding quality than DA INT in CA1, while having similar time lags, precision and reliability timescales.

Animal ID	Opsins	Probe	Light sources	Sessions	PYR	INT	AC response PYR	AC response INT
**Neocortex**								
mC41	CaMKII::ChR2	Stark64	5x470 nm, 1x356 nm LEDs	5	119	21	42	8
mF84	CaMKII::ChR2	Linear32	1x470 nm LED	5	48	19	4	4
mF93	CaMKII::ChR2	Stark64	5x470 nm LEDs	3	161	87	26	9
mF108	CaMKII::ChR2	Stark64	4x470 nm LEDs	2	75	62	31	26
mA234	CaMKII::ChR2	Buzsaki32	3x470 nm LEDs	11	359	132	62	21
mDS1	CaMKII::ChR2	Dual-sided64	2x470 nm LEDs	5	46	31	26	5
mDS2	CaMKII::ChR2	Dual-sided64	2x470 nm LEDs	8	1059	211	358	91
mDL5	PV::ChR2	Buzsaki32	2x470 nm LEDs, 1x450 nm LDs	14	159	62	0	23
mP77	PV::ChR2	Buzsaki32	2x470 nm LEDs	5	26	50	0	7
mP151	PV::ChR2	Buzsaki32	2x470 nm LEDs	7	44	25	0	4
mP20	PV::ChR2	Stark64	6x470 nm LEDs	4	97	40	0	5
mP31	PV::ChR2	Stark64	4x470 nm LEDs	8	432	103	0	27
**CA1**								
mC41	CaMKII::ChR2	Stark64	5x470 nm, 1x356 nm LEDs	21	1535	265	345	65
mDS1	CaMKII::ChR2	Dual-sided64	2x470 nm LEDs	10	397	33	106	11
mDS2	CaMKII::ChR2	Dual-sided64	2x470 nm LEDs	3	126	57	78	19
mA234	CaMKII::ChR2	Buzsaki32	3x470 nm LEDs	21	1299	180	126	58
mP23	PV::ChR2	Buzsaki32	2x470 nm LEDs, 2x450 nm LDs	2	36	8	0	3
mP101	PV::ChR2	Buzsaki32	4x470 nm LEDs	6	159	29	0	7
mP151	PV::ChR2	Buzsaki32	2x470 nm LEDs	12	27	30	0	11
mP30	PV::ChR2	Stark64	4x470 nm LEDs	3	132	30	0	0
mP31	PV::ChR2	Stark64	4x470 nm LEDs	7	280	85	0	9

Table S1. Unit yield from every experimental animal. Related to STAR Methods.
