# Supplementary material for: Error correction and improved precision of spike timing in converging cortical networks
Source: Cell Rep. 2022 Sep 20;40(12):111383. doi: 10.1016/j.celrep.2022.111383 (PMC9513803; doi:10.1016/j.celrep.2022.111383)
Supplement: Document S2. Article plus supplemental information [file mmc2.pdf]

Cell Reports

Article

Error correction and improved precision of spike timing in converging cortical networks

Graphical abstract

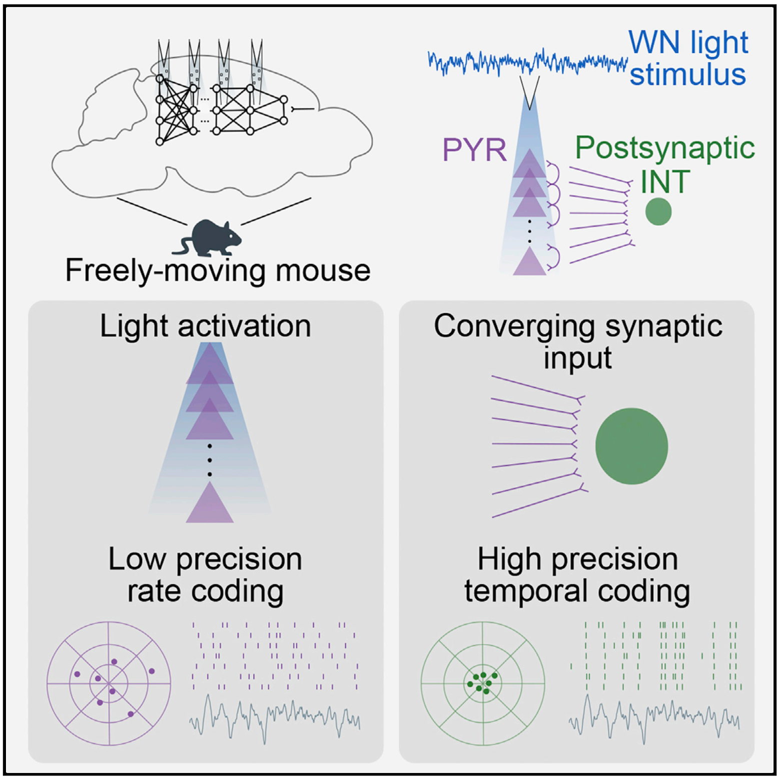

Highlights

- PYR-to-interneuron spike transmission exhibits error correction and improved precision
- Interneuron precision is higher when a larger pool of presynaptic PYRs is recruited
- Error correction and improved precision are consistent with coincidence detection
- Interneurons activated by synaptic transmission act as temporal coders

Authors

Amir Levi, Lidor Spivak, Hadas E. Sloin, Shirly Someck, Eran Stark

Correspondence

eranstark@tauex.tau.ac.il

In brief

Using optogenetic injection of white noise to pyramidal cells in neocortex and CA1 of freely moving mice, Levi et al. investigate mechanisms that enable the precise propagation of messages to postsynaptic interneurons. They find that postsynaptic interneurons act as temporal coders, exhibiting higher spike-timing precision than the neurons directly activated by the white noise signal.

Levi et al., 2022, Cell Reports *40*, 111383
September 20, 2022 © 2022 The Author(s).
https://doi.org/10.1016/j.celrep.2022.111383

CellPress

Cell Reports

CellPress
OPEN ACCESS

Article

Error correction and improved precision of spike timing in converging cortical networks

Amir Levi,[1] Lidor Spivak,[1] Hadas E. Sloin,[1] Shirly Someck,[1] and Eran Stark[1,2,*]
[1]Sagol School of Neuroscience and Department of Physiology and Pharmacology, Sackler Faculty of Medicine, Tel Aviv University, Tel Aviv 6997801, Israel
[2]Lead contact
*Correspondence: eranstark@tauex.tau.ac.il
https://doi.org/10.1016/j.celrep.2022.111383

SUMMARY

The brain propagates neuronal signals accurately and rapidly. Nevertheless, whether and how a pool of cortical neurons transmits an undistorted message to a target remains unclear. We apply optogenetic white noise signals to small assemblies of cortical pyramidal cells (PYRs) in freely moving mice. The directly activated PYRs exhibit a spike timing precision of several milliseconds. Instead of losing precision, interneurons driven via synaptic activation exhibit higher precision with respect to the white noise signal. Compared with directly activated PYRs, postsynaptic interneuron spike trains allow better signal reconstruction, demonstrating error correction. Data-driven modeling shows that nonlinear amplification of coincident spikes can generate error correction and improved precision. Over multiple applications of the same signal, postsynaptic interneuron spiking is most reliable at timescales ten times shorter than those of the presynaptic PYR, exhibiting temporal coding. Similar results are observed in hippocampal region CA1. Coincidence detection of convergent inputs enables messages to be precisely propagated between cortical PYRs and interneurons.

INTRODUCTION

During sensory processing, cognitive thought, and action generation, spiking neuronal signals are propagated accurately and rapidly across multiple brain regions (Buračas et al., 1998). However, information transmission may be accompanied by increased variability (Kara et al., 2000). Understanding the mechanisms underlying the precise and reliable propagation of spiking signals between neurons is an open question in neuroscience (Sadeh and Clopath, 2021). Any signal processing can only induce distortion (Cover and Thomas, 1991). In particular, the propagation of spikes via synaptic transmission can only add noise in the form of lost spikes, added background activity, or spike time jitter, requiring a neuronal mechanism to minimize and compensate for added noise. Perhaps the simplest mechanism for precise and reliable propagation of spiking signals is 1:1 transmission between a source and a target neuron. Indeed, some neurons generate postsynaptic responses that are sufficiently strong to generate postsynaptic action potentials (Henze et al., 2002; Szegedi et al., 2017). In particular, pyramidal cells (PYRs) may connect to neighboring neurons with millivolt-scale unitary excitatory postsynaptic potentials (EPSPs) (Cossell et al., 2015; Pala and Petersen, 2015). However, 1:1 transmission cannot compensate for added noise. Furthermore, cortical spike transmission is typically probabilistic and weak: most unitary EPSPs fail to generate spikes in postsynaptic neurons (Galarreta and Hestrin, 2001; Jouhanneau et al., 2018).

Another possible mechanism for propagating spiking signals is divergence to multiple presynaptic neurons, which may in turn converge on a single postsynaptic target to generate a response (Griffith, 1963). Transmission via convergent inputs has been explored in theoretical models (Abeles, 1991; Rothman et al., 1993; Diesmann et al., 1999), *in vitro* (Reyes, 2003; Xu-Friedman and Regehr, 2005; Rossant et al., 2011), in non-mammalian systems (Bhandawat et al., 2007; Jeanne and Wilson, 2015), and in early stages of sensory processing (Joris et al., 1994; Kuenzel et al., 2011). However, transmission via convergence has not been demonstrated for cortical circuitry in the intact brain. Thus, it is still unclear whether anatomical convergence (Braitenberg and Schüz, 1998) and weak connectivity are sufficient to convey a spike pattern precisely and reliably to a postsynaptic cell.

To determine the precision and reliability timescale of spike transmission in converging neuronal networks, we focused on the interface between PYRs and inhibitory interneurons (INTs; mostly fast-spiking putative parvalbumin-immunoreactive [PV] cells) in association cortices (parietal cortex and hippocampal region CA1) of freely moving mice. Although information may propagate between excitatory cells, the PYR-to-INT interface provides a convenient model system since connectivity is relatively easy to detect and transmission is largely self-terminating due to the inhibitory nature of the INTs. INTs assist in curtailing runaway excitation (Yizhar et al., 2011), shaping local circuit function (Pouille and

This is an open access article under the CC BY license (http://creativecommons.org/licenses/by/4.0/).

Scanziani, 2001), and propagating information (Stark et al., 2013). In both PYRs (Galarreta and Hestrin, 2001) and INTs (Bacci and Huguenard, 2006), spikes can be generated with millisecond timescale precision. As input, we used filtered Gaussian white noise (WN) signals, composed of a rich set of waveforms and spectral components. Previous studies employed WN signals to study stimulus-response properties (De Boer and Kuyper, 1968; Eggermont et al., 1983; Bialek et al., 1991; Reid et al., 1997; DiCarlo et al., 1998), spike generation (Bryant and Segundo, 1976; Mainen and Seinowski, 1995; Billimoria et al., 2006), and spike transmission (Marmarelis and Naka, 1972). We applied the same WN signal multiple times to the same set of PYRs using optogenetic activation, and determined the precision and reliability timescale of the directly activated PYRs (DA PYRs) and of postsynaptic, indirectly activated INTs (IDA INTs).

RESULTS

Determining spiking precision and reliability from the response to filtered Gaussian white noise

At least two temporal properties of single-neuron spike trains should be distinguished: (1) how precise spike timing is with respect to an input signal, and (2) at what timescale spike timing is most consistent over multiple presentations of the same signal. In the intact brain, ongoing spiking (“noise”) occurs even when a “signal” (e.g., the WN pattern) is applied. To rigorously define precision and reliability timescale in the presence of noise, we proceeded as follows. First, we generated a cross-validated filter based on the spike-triggered average of the WN signal (a modified Wiener filter; STAR Methods), and used the filter to reconstruct the input from the spike trains (Figure 1A). The peak of the filter defines the time lag between the spike train and the input (Figure 1A, inset). The rank correlation coefficient (cc) between the input and the reconstructed signals, averaged over all trials, defines decoding quality Q (Figure 1B, top). We defined “precision” P as the temporal jitter (half-width of the jittering interval, δ) at which Q begins degrading (Figure 1B). The choice of the specific filter model (Figures S1A–S1D; STAR Methods) does not affect any of the definitions, steps, or conclusions (Figures S1E–S1I). Second, we defined “reliability timescale” Rp as the temporal variance (SD of a Gaussian kernel, σ) at which spike trains are most similar to one another (Figure 1C). Hereafter, a set of spike trains recorded during repeated presentation of the same input signal is assigned two numbers: P and Rp. In artificial spike trains designed to imitate spiking response in the presence of noise, precision depends on signal jitter (Figure 1D), whereas reliability timescale depends on noise jitter (Figure 1E). Large values of P indicate poor precision (Figures 1F and 1G), whereas small values indicate high precision (Figures 1H and 1I). Smaller values of Rp (Figures 1F and 1H) are consistent with highest reliability at shorter timescales, whereas larger values (Figures 1G and 1I) indicate that reliability timescale is longer. From the perspective of a downstream target, spike trains with short reliability timescales can be read out at higher temporal resolutions (“temporal” codes; Pouille and Scanziani, 2001), whereas long-timescale trains require integration over longer windows (“rate” codes; Barlow, 1972; London et al., 2010). Spike trains with short/long reliability timescales can exhibit high/poor precision (Figure 1J), across a wide range of firing rates (Figure 1K). Thus, precision and reliability timescale are dissociable.

Spike transmission between PYRs and INTs exhibits error correction and improved precision

To measure distortion over the PYR-to-INT interface, we used WN-shaped optogenetic signals to focally activate PYRs in the parietal association cortex of freely moving mice (Figure 2A). We used microwatt-scale light power, avoiding induction of field oscillations (Stark et al., 2014). We then quantified decoding quality and precision for DA PYRs (presynaptic) and for IDA INTs (postsynaptic) with respect to the WN input signal applied only to the DA PYRs. In an example session, we recorded 134 well-isolated PYRs and INTs (Figure 2B). Over 200 presentations of the same WN signal, a given DA PYR spiked at different times (Figure 2C, top), and different PYRs exhibited various precision values (1.8–6.8 ms; Figure 2B). A simultaneously recorded postsynaptic INT responded with higher precision than all DA PYRs (0.4 ms; Figure 2C, bottom). Thus, an IDA INT may exhibit higher precision than a DA PYR.

We recorded 1,867 neocortical PYRs and 563 INTs from n = 7 CaMKII::ChR2 mice (Table S1). We defined a reliable response to the WN signal whenever the firing rate increase, the decoding quality, and the reconstruction-based reliability were all significant (Figures S2A–S2C; STAR Methods). Overall, 239 DA PYRs and 116 IDA INTs exhibited reliable responses. Modified Wiener filters for DA PYRs and IDA INTs peaked at negative time lags (Figure 2D), but DA PYR filters peaked closer to spike time (Figure 2E). For DA PYR, the median time lag was 2.4 ms (Figure 2F). Simultaneously recorded IDA INTs exhibited longer time lags (4 ms; $p < 0.001$, U test; Figure 2F). The 1.6 ms time lag difference is not consistent with direct optical activation of the INT and is rather consistent with synaptic INT activation. Instead of being distorted by transmission, decoding quality for IDA INTs (median [interquartile interval; IQR] Q = 0.19 [0.12 0.27]) was higher than for DA PYRs (Q = 0.13 [0.11 0.18]; $p < 0.001$, U test; Figure 2G). Similar results were observed for decoding measured by mutual information ($p < 0.001$, U test; Figures S2D–S2F) and spectral coherence ($p = 0.0013$, U test; Figures S2G–S2J). Thus, the WN signal applied to the PYRs is reconstructed better based on the postsynaptic compared with the presynaptic spike trains. We term this phenomenon “error correction.”

The median [IQR] precision of the DA PYRs was 3.2 [2.4 4] ms (Figure 2H). Therefore, neocortical PYRs can emit spikes with precision of a few milliseconds with respect to rapidly changing inputs, extending previous *in vitro* observations (Mainen and Seinowski, 1995). In contrast, IDA INT precision was 2.3 [1.6 3.2] ms, higher than DA PYR precision ($p < 0.001$, U test; Figure 2H). Thus, with respect to the WN signal applied to the PYRs, spiking precision of postsynaptic INTs is higher than the precision of the presynaptic PYRs. We term this phenomenon “improved precision.”

To identify what determines precision, we considered a host of single-neuron properties that together accounted for most group variance (R^2 = 0.7; Figure S3). DA PYR precision was not correlated with DA PYR firing rate during baseline (0.7

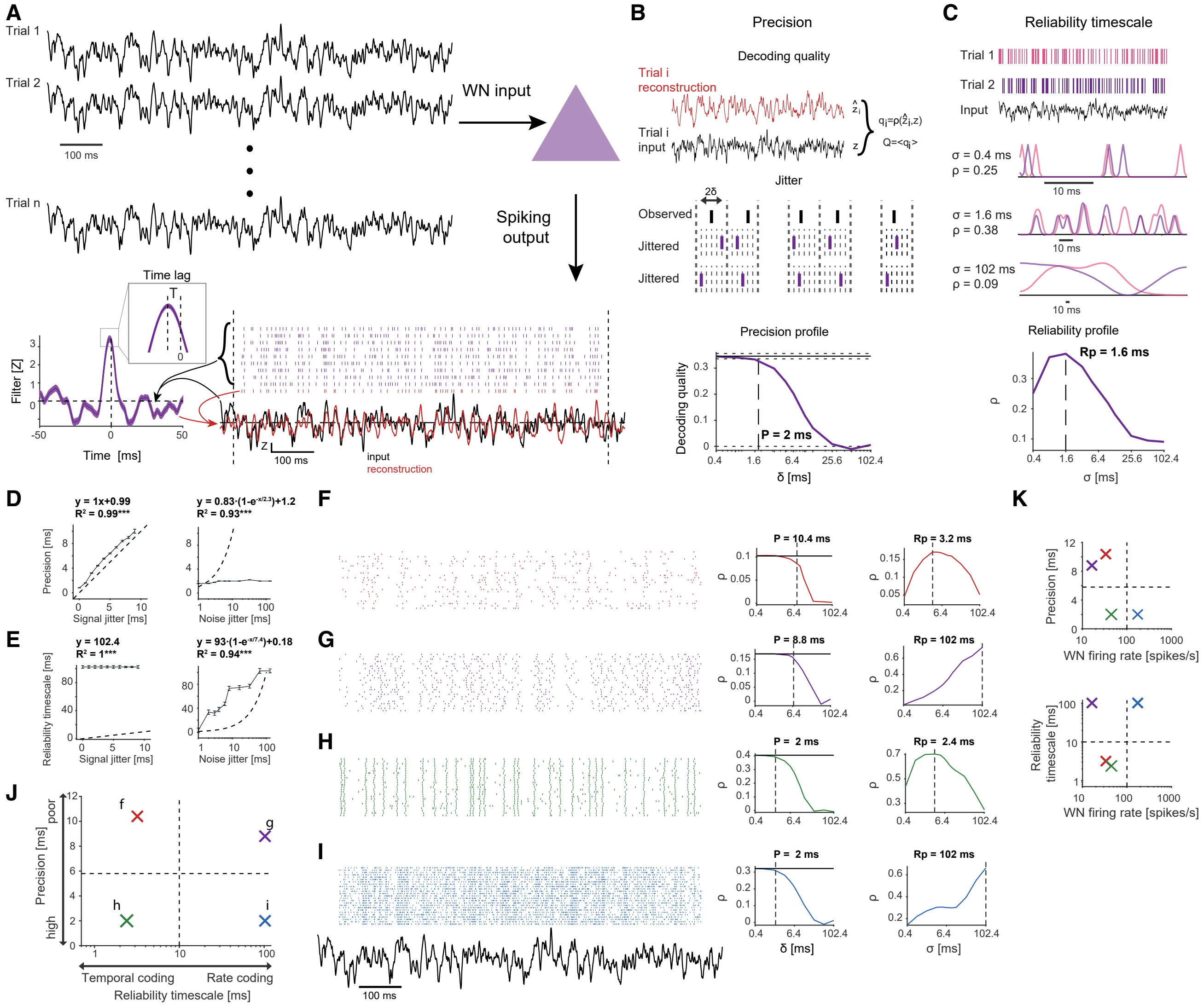

Figure 1. Dissociating spiking precision and reliability timescale

(A) Cross-validated reconstruction. Input is applied as a WN signal. An acausal modified Wiener filter model is generated, based on the WN and training-set spike trains (purple). The model is used to reconstruct the WN signal during test-set spike trains (red). Dashed lines delimit spikes used for filter generation.

(B) Decoding quality Q is defined as the trial-averaged cc between the WN input and the reconstructed signals. Interval jitter is used to determine precision P. Every spike is jittered within a 2δ ms window and Q is recomputed. Precision is defined as the δ for which the jittered Q is consistently below the original Q (Wilcoxon test, $p < 0.05$).

(C) Reliability is the cc between pairs of reconstructed trials, averaged over all possible pairs. To determine reliability timescale (Rp), reconstructions are based on a discrete set of Gaussian kernels with distinct SDs, resulting in the reliability profile (bottom).

(D) In artificial data, when noise jitter is held constant at zero, precision is a monotonically increasing function of signal jitter (left here and in (E), ***$p < 0.001$, F test). Dashed lines indicate unity.

(E) When noise jitter is held constant at zero, reliability timescale is constant (left). When signal jitter is held constant, reliability timescale is a monotonically increasing function of noise jitter (right).

(F–I) Artificial examples with (F) poor precision, short reliability timescale; (G) poor precision, long reliability timescale; (H) high precision, short reliability timescale; and (I) high precision, long reliability timescale.

(J) A monotonically increasing reliability profile is denoted a "rate coder" (G, I). A unit with a reliability profile with a consistent peak at an SD other than the maximally tested SD is denoted a "temporal coder" (F, H). High precision can be achieved for both temporal and rate coders.

(K) During WN trials, rate/temporal coding (top) and high/poor precision (bottom) can be achieved for higher and lower firing rates. See also Figure S1.

[0.24 1.73] spikes/s; cc = −0.09; p = 0.24, permutation test). However, precision was higher when WN firing rate was higher (partial cc −0.2; $p < 0.001$, permutation test; Figure S3J). IDA INTs exhibited higher WN firing rates than DA PYRs (median 28 versus 12 spikes/s; $p < 0.001$, U test; Figure S3O). To determine whether IDA INT decoding quality and precision

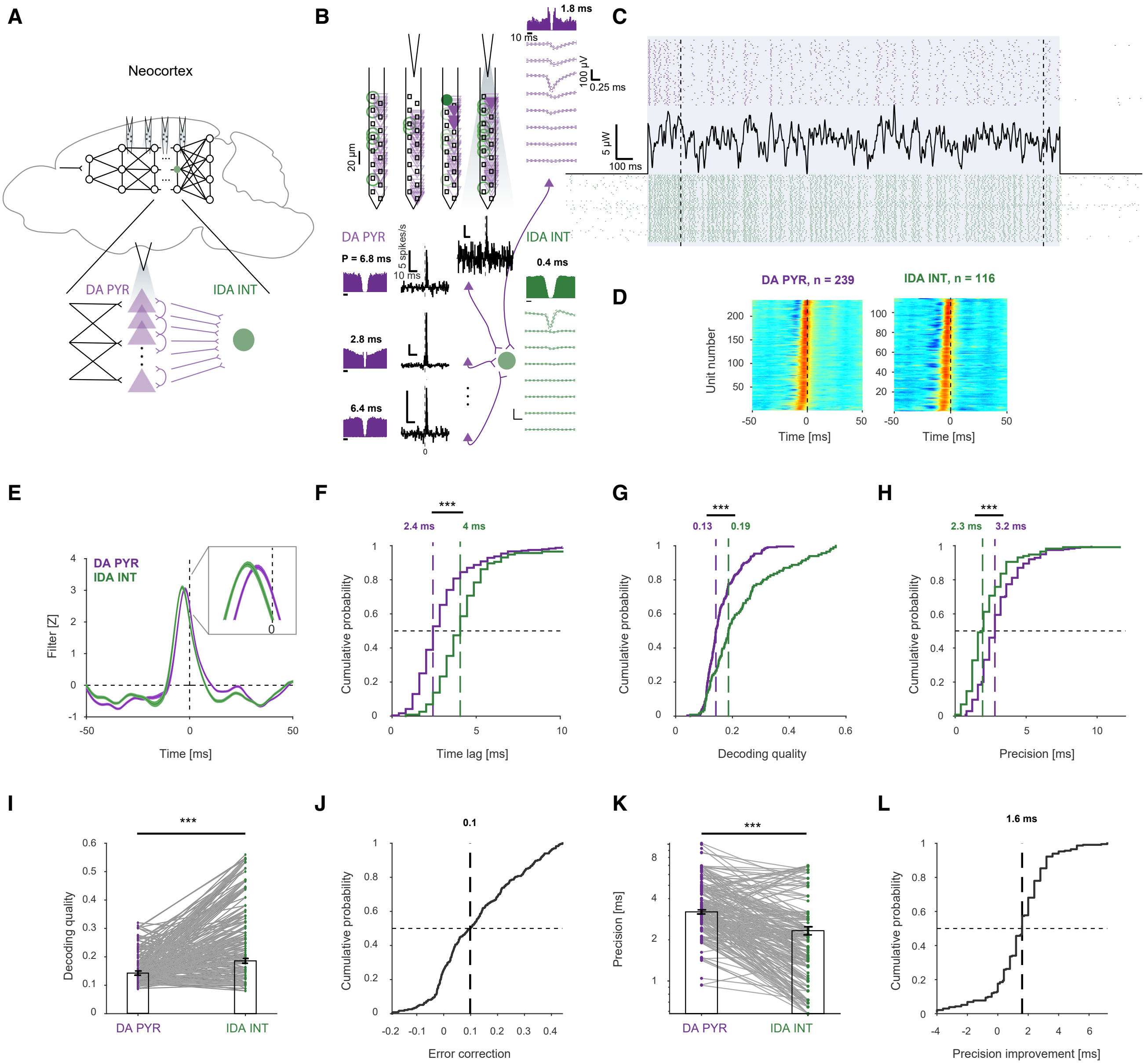

Figure 2. Error correction and improved precision along the PYR-to-INT interface

(A) WN was applied to groups of neocortical DA PYRs using optogenetic illumination.

(B) Subnetwork of four PYRs (purple) and one putative postsynaptic INT (green), recorded simultaneously (of 112 PYRs and 22 INTs) using a high-density optoelectronic probe. Auto-correlation histograms (ACHs) and cross-correlation histograms (CCHs; no light condition), consistent with monosynaptic excitation ($p < 0.001$, Poisson test).

(C) Spike trains of one PYR (top) and INT (bottom) during 50 (of 200) WN trials.

(D) Stacked filters for DA PYRs (left) and IDA INTs (right; n = 7 CaMKII:ChR2 mice).

(E) Mean ± SEM filters for the DA PYR and IDA INT populations. The DA PYR filter peak occurs closer to the spike than the IDA INT filter peak.

(F) DA PYR short time lags indicate direct activation, whereas longer IDA INT time lags imply indirect (i.e., synaptic) activation. Here and in (G), (H), (J), and (L), vertical dashed lines indicate median values; ***$p < 0.001$, U test.

(G) As a population, IDA INTs have higher decoding quality than DA PYRs.

(H) IDA INT spike trains are more precise than DA PYR trains.

(I) In 174/229 (76%) connected pairs, IDA INT decoding quality is higher than presynaptic DA PYR decoding quality. Here and in (K), ***$p < 0.001$, Wilcoxon test.

(J) Error correction, defined as decoding quality difference between connected DA PYR/IDA INT pairs.

(K) In 191/229 (84%) connected pairs, IDA INT precision is higher than presynaptic DA PYR precision.

(L) Precision improvement between connected pairs. See also Figures S2 and S3.

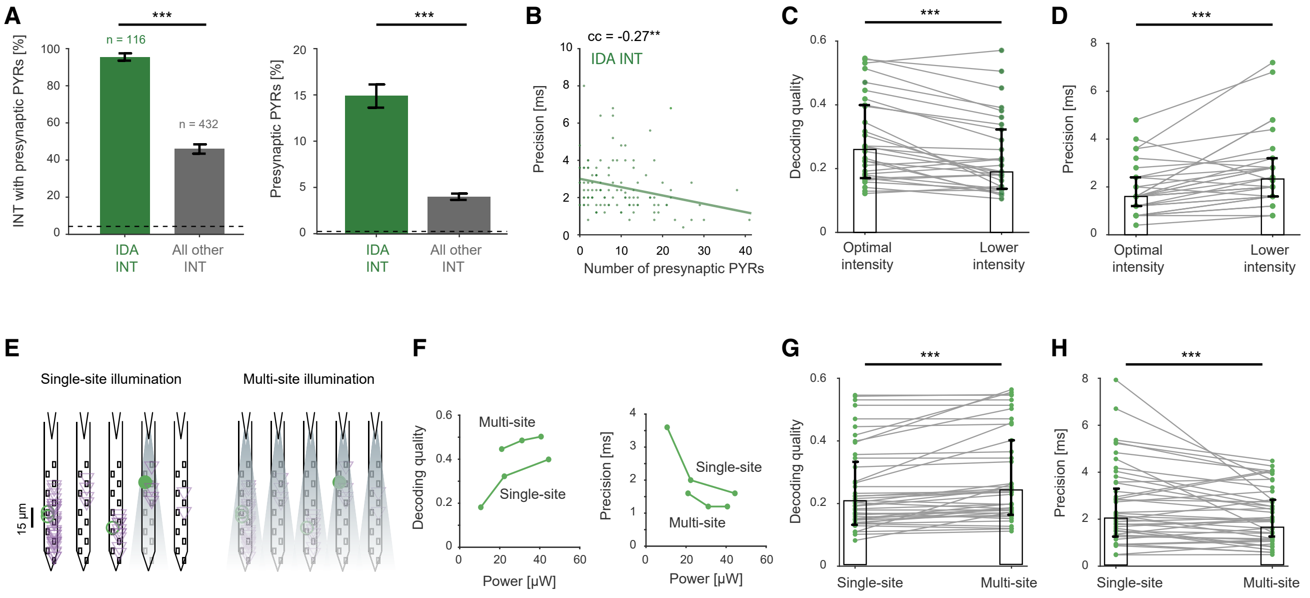

Figure 3. Postsynaptic decoding quality and precision depend on presynaptic assembly size

(A) Network connectivity is denser for IDA INTs than for simultaneously recorded INTs not activated by the WN signal. Left: IDA INTs are more likely to have at least one recorded presynaptic PYR (***$p < 0.001$, G test of independence; dashed line indicates chance level). Right: probability of local connectivity is higher for IDA INTs (***$p < 0.001$, U test).

(B) IDA INT precision is correlated with the number of recorded presynaptic DA PYRs (**$p < 0.01$, permutation test).

(C and D) When multiple intensities were used (n = 34 IDA INTs), the optimal light intensity was denoted as the intensity that yielded the highest DC gain. IDA INTs tested during lower intensity exhibit decreased decoding quality (C) and poorer precision (D). Here and in (G) and (H), ***$p < 0.001$, Wilcoxon test.

(E and F) Example experiment. WN signals were applied to neocortical PYRs using either single-site or multisite illumination. For the example IDA INT (E, green circle), decoding quality (F, left) and precision (F, right) increased when multisite illumination was used.

(G and H) IDA INTs tested during single- and multisite illumination of similar intensities (n = 55) exhibit increased decoding quality (G) and higher precision (H) during multisite illumination.

depend on WN firing rates, we pruned IDA INT trains to match the DA PYR rates by removing the spikes least likely to have emanated from the WN input. The procedure did not improve IDA INT decoding quality or modify error correction (Figures S3R and S3S). Yet even after pruning, IDA INT precision was higher than DA PYR precision ($p < 0.001$, U test; Figures S3T and S3U). Thus, error correction and improved precision are maintained regardless of firing rates.

In 174/229 (76%) of the putatively connected monosynaptic PYR-to-INT pairs, IDA INT decoding quality was higher than decoding quality of the presynaptic DA PYRs ($p < 0.001$, Wilcoxon test; Figure 2I), exhibiting error correction. Error correction was higher on a pair-by-pair basis (Figure 2J) than at the population level ($p < 0.001$, permutation test). Furthermore, in 191/229 (84%) of the monosynaptic PYR-to-INT pairs, IDA INT precision was higher than DA PYR precision ($p < 0.001$, Wilcoxon test; Figure 2K). The improvement in precision was higher on a pair-by-pair basis (median 1.6 ms; Figure 2L) than at the population level (3.2–2.3 = 0.9 ms; $p < 0.001$, permutation test). In sum, error correction and improved precision are observed following PYR-to-INT spike transmission.

Postsynaptic decoding quality and precision depend on presynaptic assembly size

Since IDA INTs are activated by DA PYRs, IDA decoding quality may depend on presynaptic connectivity. Indeed, 109/116 (94%) IDA INTs had at least one presynaptic PYR (median [IQR]: 8 [4 14]), whereas only 207/432 (48%) of the simultaneously recorded INTs not driven by the WN were connected to PYRs ($p < 0.001$, U test; Figure 3A, left). Furthermore, IDA INT connection probability was higher (15% versus 4%; $p < 0.001$, U test; Figure 3A, right). Thus, compared with simultaneously recorded INTs not driven by WN, network connectivity is denser for IDA INTs.

To determine whether postsynaptic decoding quality and precision depend on presynaptic assembly size, we first quantified correlations. We found that IDA INT decoding quality did not depend consistently on the number of recorded DA PYRs (cc = 0.17; p = 0.08, permutation test). In contrast, IDA INT precision was higher when more DA PYRs were recorded (cc = −0.27, p = 0.002; Figure 3B). To determine causally whether postsynaptic decoding quality and precision depend on the number of presynaptic PYRs, we carried out two targeted experiments. First, we tested the effect of reducing presynaptic pool size by comparing IDA INT decoding quality when WN signals were applied at the optimal light intensity and at lower intensity (median [IQR] ratio: 0.49 [0.48 0.5]). Reducing the intensity reduced postsynaptic decoding quality (ratio: 0.8 [0.7 1]; n = 34 IDA INTs; $p < 0.001$, Wilcoxon test; Figure 3C) and impaired postsynaptic precision (ratio: 1.5 [1.2 2]; $p < 0.001$; Figure 3D). Next, we tested the effect of increasing presynaptic pool size by multi-site illumination, without

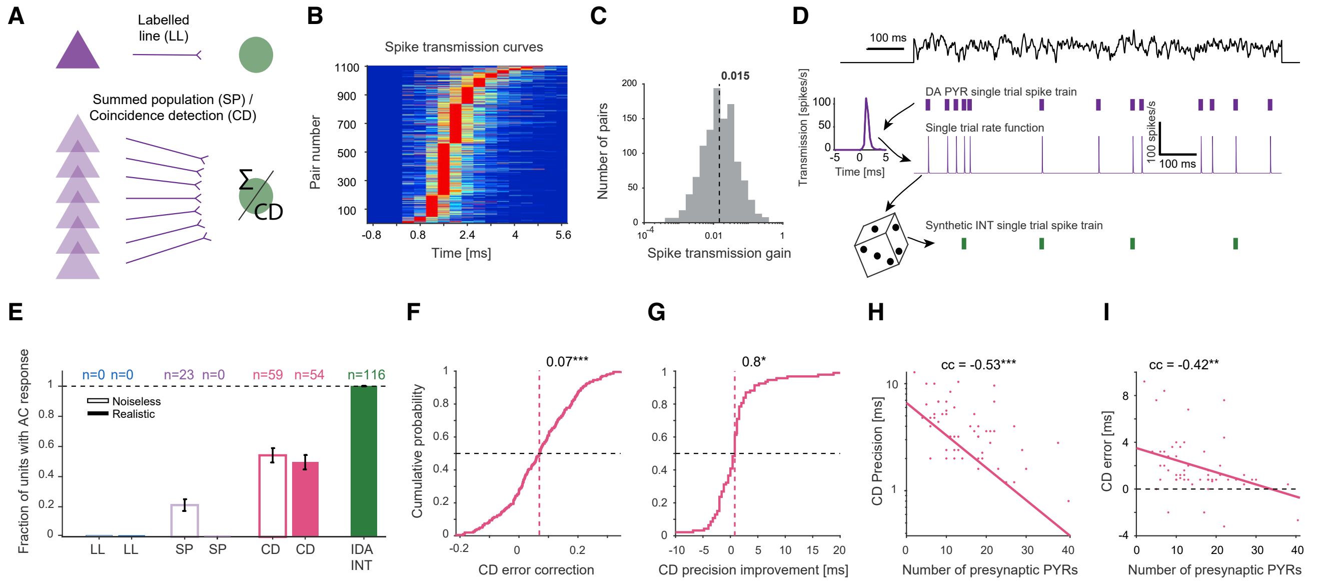

Figure 4. Error correction and improved precision are consistent with coincidence detection

(A) Three transmission models were used for converting spike trains of one (top, LL) or multiple simultaneously recorded (bottom, SP/CD) presynaptic DA PYR spike trains into an sINT train.

(B) Spike transmission curves for all PYR to IDA INT pairs with monosynaptic connections (1,106 pairs).

(C) Spike transmission gain, defined for every pair of units as the area under the spike transmission curve.

(D) Recorded DA PYR spikes (middle, purple ticks) during every individual WN trial (top, black trace) were convolved with the spike transmission curve extracted from the CCH (left), yielding a single trial rate function (middle, purple trace). A noiseless sINT train (bottom, green ticks) was stochastically generated from the rate function. A realistic sINT train was generated by adding "background" spikes randomly, equalizing the firing rates of the sINT and the postsynaptic IDA INT.

(E) Under noisy conditions, only CD sINTs exhibit AC responses.

(F and G) sINTs exhibit error correction (F) and improved precision (G); *p < 0.05 and ***p < 0.001, Wilcoxon test.

(H) sINT precision is correlated with the number of recorded presynaptic DA PYRs. Here and in (I): **p < 0.01 and ***p < 0.001, permutation test.

(I) CD error, the difference between the precision of the CD sINTs and the corresponding IDA INTs. See also Figure S4.

modifying single-source light power (Figures 3E and 3F). Compared with single-site illumination, multi-site illumination induced higher IDA INT decoding quality (ratio: 1.1 [1 1.3]; n = 55 IDA INTs; $p < 0.001$; Figure 3G) and improved the precision (ratio: 0.9 [0.7 1]; $p < 0.001$; Figure 3H). Thus, when a larger presynaptic assembly is recruited, IDA INT decoding quality and precision are higher.

Error correction and improved precision are consistent with coincidence detection

Precise transmission of spikes from a presynaptic source to a postsynaptic target can be achieved by at least three conceptually distinct models (Figure 4A). A single presynaptic neuron may drive the target strongly via a "labeled line" (LL; Henze et al., 2002; Szegedi et al., 2017). Second, multiple simultaneously active neurons may generate EPSPs that sum linearly in the postsynaptic neuron ("summed population"; SP; Cash and Yuste, 1999; Tran-Van-Minh et al., 2016). Finally, simultaneously active neurons may generate supralinear postsynaptic effects due to dendritic and/or somatic nonlinearities ("coincidence detection"; CD; Azouz and Gray, 2000; Stuart and Häusser, 2001; Polsky et al., 2004; Ferrarese et al., 2018). To examine model plausibility, we generated synthetic postsynaptic spike trains (sINTs) based on the recorded PYR trains and data-dependent filters (Figures 4B–4D). When background spiking was included, the LL model (Figures S4A–S4D) and the SP model (Figures S4E–S4I) did not yield any reliably responding sINTs (Figure 4E).

In contrast, the CD model (Figure S4J–S4T) yielded 54/109 (50%) driven sINTs (Figure 4E). sINT precision was poorer than actual IDA INT precision ($p < 0.001$, Wilcoxon test; Figure S4N). However, the precision of the CD-driven sINTs was higher than the precision of the corresponding presynaptic DA PYRs by 0.8 ms (median; p = 0.03, Wilcoxon test), exhibiting improved precision on a pair-to-pair basis (Figure 4G). Furthermore, the CD-driven sINTs exhibited error correction ($p < 0.001$, Wilcoxon test; Figure 4F). These results depended on the extent of nonlinearity, but were independent of implementation specifics (multiplicative or exponential nonlinearity; Figures S4P–S4T). Since all data-driven models are constrained by the available data, we determined what limits the CD model by quantifying performance as a function of presynaptic pool size. The precision of the CD-driven sINTs was higher when more presynaptic PYRs were recorded (cc −0.53, $p < 0.001$, permutation test; Figure 4H). Furthermore, sINT precision was increasingly more similar to the precision observed for the IDA INTs when more presynaptic PYRs were recorded (cc between CD error and number of presynaptic PYR: −0.42, p = 0.003, permutation test; Figure 4I). To conclude, while no model provides an exact match, CD yields the behavior closest to the experimental observations.

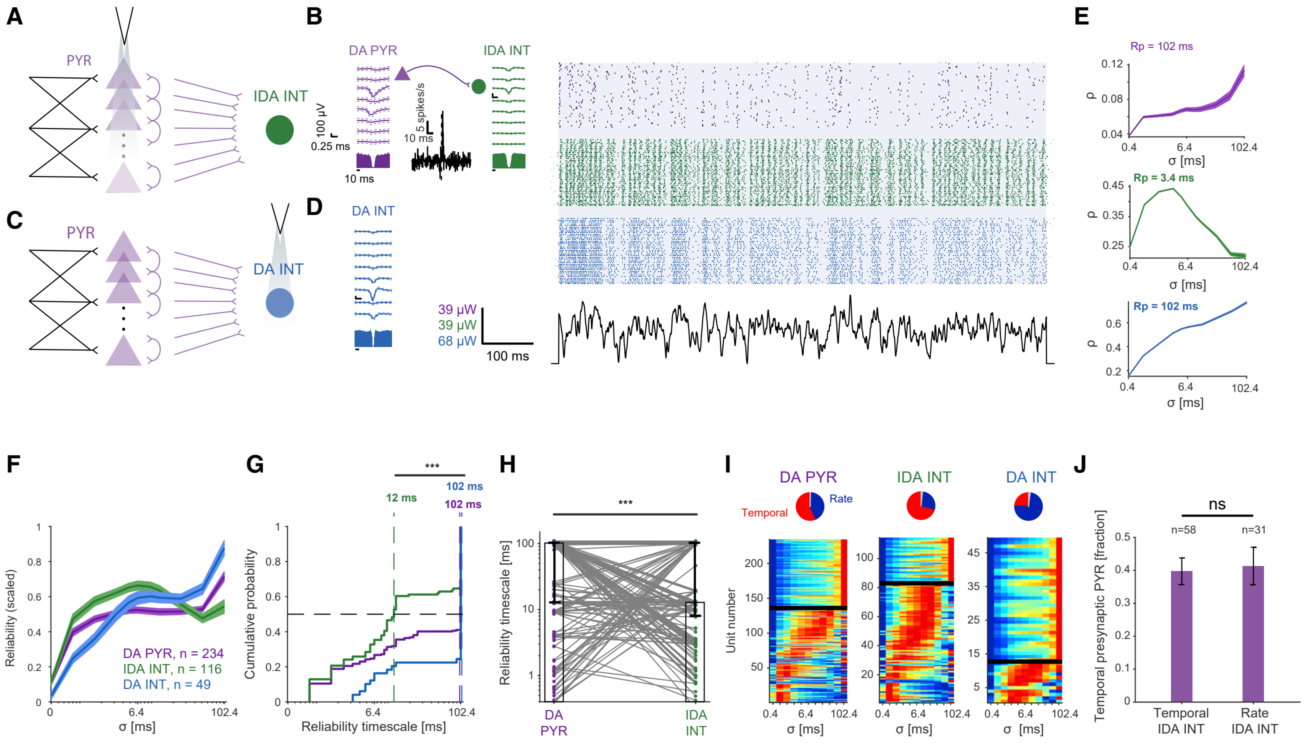

Figure 5. INTs activated by synaptic transmission act as temporal coders

(A) WN signals were applied to groups of neocortical DA PYRs (CaMKII::ChR2 mice).

(B) Example PYR (purple) and INT (green) spike trains. The CCH is consistent with monosynaptic excitation ($p < 0.001$, Poisson test). Here and in (D), all conventions are the same as in Figures 2B and 2C.

(C) Neocortical PV cells were directly activated (DA INTs; n = 5 PV::ChR2 mice).

(D) Example DA INT spike trains.

(E) The reliability profiles of the DA PYRs and DA INTs are monotonically increasing (purple and blue), whereas the IDA INTs have a shorter reliability timescale (green). All conventions are the same as in Figures 1F–1I.

(F) Averaged reliability profiles of the three populations (mean ± SEM over all scaled profiles). At the group level, IDA INTs are temporal coders.

(G) Cumulative distribution functions (CDFs) of reliability timescales of units from the three populations. Vertical dashed lines show group medians; ***$p < 0.001$, U test. IDA INTs exhibit shorter timescales than DA PYRs and DA INTs.

(H) IDA INTs have shorter reliability timescales than presynaptic DA PYRs. Gray lines indicate inferred monosynaptic connectivity. Each IDA INT timescale is plotted along with the timescales of all corresponding presynaptic DA PYRs (221 pairs); ***$p < 0.001$, Wilcoxon test.

(I) Reliability profiles of all neocortical DA PYRs, IDA INTs, and DA INTs, scaled to the 0–1 range and sorted according to peak timescale. Profiles are arranged with temporal/rate coders below/above black lines.

(J) Temporal coding of IDA INTs is unlikely to be inherited from presynaptic DA PYRs. Fractions of temporal presynaptic DA PYRs are not consistently different for temporal IDA INTs and rate IDA INTs ($p = 0.88$, G test). See also Figures S5 and S6.

INTs activated by synaptic transmission act as temporal coders

We found that improved precision depends on the number of neurons in the presynaptic pool and can be generated by nonlinear amplification of coincident presynaptic spikes. If synaptic inputs are coincident and consistent over multiple trials, IDA INT trains may exhibit short reliability timescales (“temporal coding”; Figure 5). Indeed, IDA INT reliability profiles peaked at intermediate σ (Figure 5E, center), and the global maximum in the IDA INT group profile was higher than adjacent minima ($p < 0.001$, U test; Figure 5F, green). DA PYRs exhibited mixed reliability profiles, peaking at the highest tested σ (Figure 5G). In contrast, IDA INT profiles peaked at shorter timescales (median 12 ms; $p < 0.001$, U test; Figure 5G). Shortening of reliability timescales was observed on a pair-by-pair basis for monosynaptic PYR-to-INT pairs ($p < 0.001$, Wilcoxon test; Figure 5H). Thus, whereas DA PYRs exhibit mixed types of reliability profiles, most IDA INTs (80/112, 71%; Figure 5I) act as temporal coders.

IDA INTs exhibited short reliability timescales regardless of the light source to IDA INT distance (Figures S5A–S5C). A minority of the IDA INTs exhibited rate coding, but the fraction of temporal coders did not differ consistently between DA PYRs presynaptic to rate and temporal IDA INTs ($p = 0.88$, G test of independence; Figure 5J; Figures S6A–S6F). Thus, temporal coding is unlikely to be inherited from a single DA PYR.

The high prevalence of postsynaptic temporal coding may be an inherent property of interneurons in general or an outcome of convergent synaptic transmission. To differentiate between the alternatives, we directly activated INTs in n = 5 PV::ChR2 mice (Figures 5C and 5D). Most DA INT reliability profiles (36/49 PV

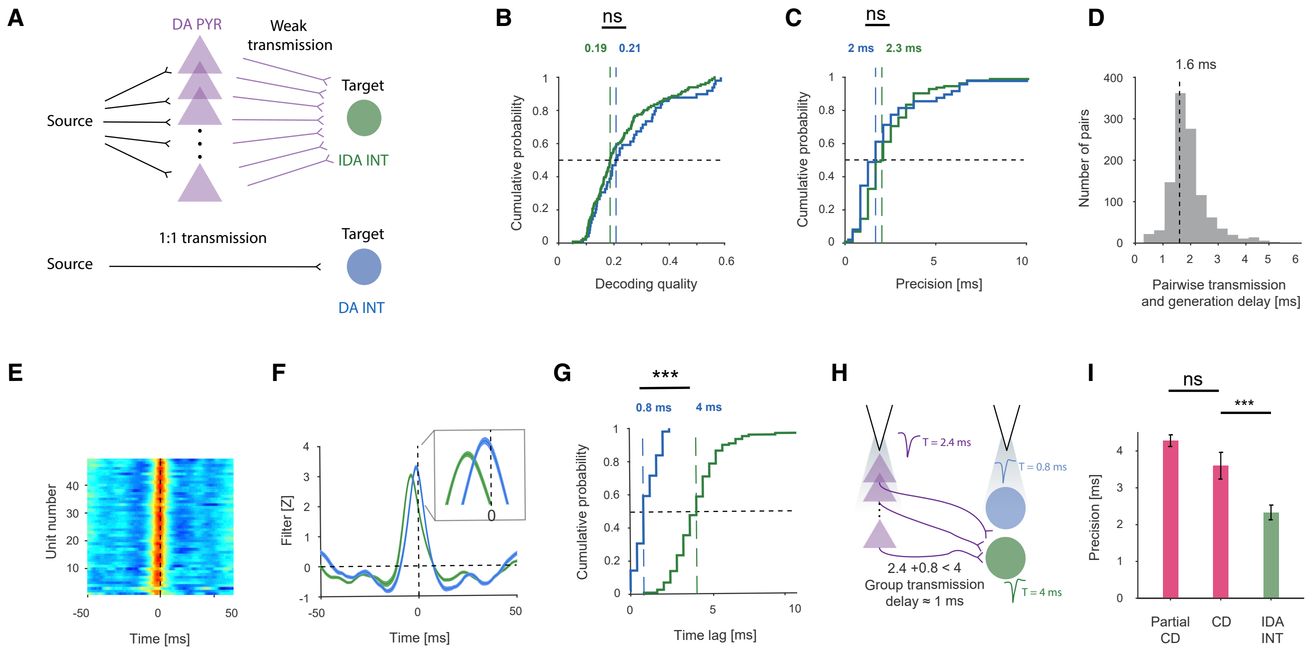

Figure 6. Convergence maintains precision and provides robustness

(A) Transmission from source to target can be achieved via a diverging/converging network (top) or direct activation (bottom).

(B) Decoding qualities of DA INTs and IDA INTs are not consistently different ($p = 0.23$, U test). Here and in (C), (D), and (G), vertical dashed lines indicate medians.

(C) Precision of DA INTs and IDA INTs is not consistently different ($p = 0.08$; U test).

(D) Transmission and generation delay of spikes over a single synaptic interface, estimated by the CCH peak.

(E) Modified Wiener filters of the DA INT population.

(F) Average (±SEM) filters for DA INTs and IDA INTs. The mean DA INT filter peaks before the IDA INT peak.

(G) Median DA INT time lag is 3.2 ms, shorter than the IDA INT time lag; ***$p < 0.001$, U test.

(H) The measured median time lags of the three populations imply that the unmeasured information transmission delay, between a group of PYRs and a single INT, is ~1 ms.

(I) Coincidence detection allows system robustness. For each IDA INT, a "partial CD" sINT was generated by randomly removing the input from one presynaptic DA PYR. The partial CD sINT population has a precision of 4.2 ms (median; ns, $p > 0.05$, and ***$p < 0.001$; U test).

cells, 73%) were consistent with rate coding (Figures 5F and 5I). Median DA INT timescales were 102 ms, consistently longer than IDA INT timescales (12 ms; $p < 0.001$, U test; Figure 5G). For the combined IDA and DA INT population, reliability timescales correlated with direct/indirect activation (partial cc −0.21; $p = 0.01$; permutation test; Figure S5N). IDA INT timescales remained shorter than DA INT timescales even when IDA INTs were pruned according to spike waveform and auto-correlation histogram (ACH) properties of DA INTs ($p < 0.001$, U test; Figures S6G–S6N). Thus, IDA INT short reliability timescales likely result from synaptic activation, as opposed to the direct optogenetic drive of the DA INTs. In summary, when INTs are activated via multiple converging inputs, temporal coding occurs.

Convergence maintains precision at the cost of a millisecond delay

There are at least two pathways for transmitting information between two stations, a "source" and a "target": direct and diverging/converging (Figure 6A). Although CD of converging inputs generates error correction and improved precision, it is unclear whether a diverging/converging configuration is advantageous, compared with a direct connection. Here, we model the source station by the WN signal and the target by INT spiking. We found that signal routing via an intermediary (the DA PYR assembly) did not consistently degrade decoding quality ($p = 0.23$, U test; Figure 6B) or precision ($p = 0.08$, U test; Figure 6C). Thus, IDA INT precision is close to the "limit" achieved by DA INTs.

The alternative to direct transmission, convergence onto a target, may involve an additional delay. The delay imposed by spike transmission and generation over a single synaptic interface can be estimated by the peak lag of the spike transmission curve (Figure 4B). For monosynaptic PYR-to-IDA INT pairs, the median lag was 1.6 ms (1,106 pairs; Figure 6D), not consistently distinct from the time lag difference estimated from the IDA INT and DA PYR reconstruction filters (4–2.4 = 1.6 ms; Figure 2F; U test, $p = 0.97$). Thus, convergence neither slows nor accelerates spike transmission. Although dynamics over a single synapse can be measured (Midorikawa and Sakaba, 2015), transmission lag over an axo-axonic interface cannot be quantified directly. Yet the lag of converging transmission can be estimated indirectly from the difference between the IDA INT time lag (PYR spike generation, PYR-to-INT transmission, and INT spike generation; 4 ms) and the sum of the DA PYR time lag (PYR spike generation; 2.4 ms) and the DA INT time lag (INT spike generation; 0.8 ms; Figures 6E–6G). After bias correction, the group

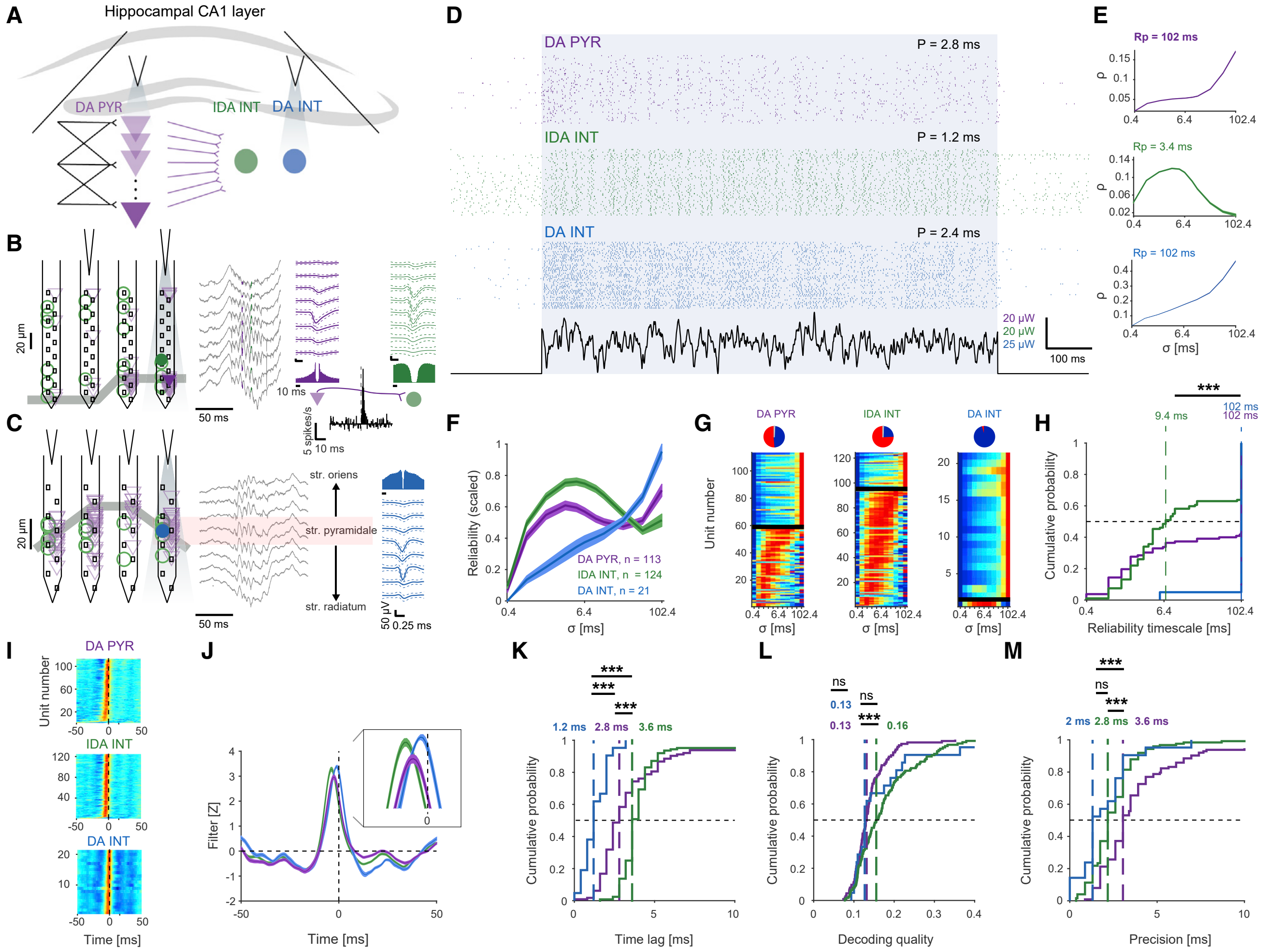

Figure 7. Temporal coding, error correction, and improved precision in CA1

(A) WN signals were applied directly to groups of PYRs or PV INTs in CA1, a hippocampal region with sparse recurrent excitation.

(B) Example DA PYRs and IDA INTs recorded in the CA1 pyramidal cell layer (CaMKII::ChR2 mouse). Center, wide-band (0.1–7,500 Hz) traces recorded by eight electrodes during a single ripple event. Here and in (C), other conventions are the same as in Figure 2B.

(C) Example DA INT recorded in CA1 (PV::ChR2 mouse).

(D) Spike trains during 50 consecutive WN trials of the three example units.

(E) The reliability profiles of DA PYR and DA INT are consistent with rate coding, whereas the IDA INT acts as a temporal coder.

(F) Reliability profiles of the three populations (mean ± SEM, scaled to the 0–1 range). At the group level, DA INTs are rate coders, IDA INTs are temporal coders, and DA PYRs exhibit mixed behavior.

(G) Reliability profiles of all DA PYRs, IDA INTs, and DA INTs in CA1.

(H) Compared with IDA INTs, DA units (PYRs and INTs) exhibit longer timescales. Here and in (K–M), vertical dashed lines show group medians; ***$p < 0.001$, U test.

(I) Stacked filters of all CA1 DA PYRs (n = 4 CaMKII::ChR2 mice), IDA INTs (recorded from the same four animals), and DA INTs (n = 5 PV::ChR2 mice).

(J) Mean ± SEM filters for the three populations.

(K) DA INTs respond faster to the WN input than DA PYRs. The longer time lags of the IDA INTs imply indirect (synaptic) activation.

(L) IDA INTs exhibit higher decoding quality than DA PYRs and DA INTs.

(M) IDA INTs have higher precision than DA PYRs. See also Figure S7.

delay was 1.02 ms (95% confidence limits 0.4–1.44 ms; Figure 6H). Thus, spike transmission over a converging synaptic interface has a millisecond timescale delay.

While indirect transmission induces an additional delay, relatively weak connections are used (median [IQR] spike transmission gain: 0.015 [0.007 0.033]; $p < 0.001$, Wilcoxon test; Figure 4C). Furthermore, indirect activation provides robustness to removal of individual intermediary elements, DA PYRs ($p = 0.95$; Figure 6I). Thus, at the cost of a millisecond delay, diverging/converging transmission allows the use of weak connections, provides robustness, and preserves precision.

Temporal coding, error correction, and improved precision in CA1

Although convergent transmission appears to support precise signal propagation along the PYR-to-INT interface, recurrent

feedback between DA PYRs may refine the process. To determine whether recurrent excitation is necessary and to generalize our findings in the neocortex, we repeated WN experiments in hippocampal CA1 (n = 4 CaMKII::ChR2 and n = 5 PV::ChR2 mice; Figures 7A–7D), a region with relatively sparse recurrent excitation (Thomson and Radpour, 1991). We found that in CA1, IDA INTs exhibited a median timescale of 9.4 ms, shorter than the DA INT timescale (102 ms; Figures 7E–7H; p < 0.001, U test). Furthermore, most IDA INTs (94/124, 76%) in CA1 acted as temporal coders, compared with 1/21 DA INTs (p < 0.001; G test of independence; Figure 7G). Thus, IDA INTs act as temporal coders regardless of the sparseness of recurrent excitation.

In CA1, the decoding quality for IDA INTs was 0.16, higher than for DA PYRs (0.13; p < 0.001, U test; Figure 7L). The precision of CA1 IDA INTs was 2.8 ms, higher than CA1 DA PYR precision (3.6 ms; p < 0.001, U test; Figure 7M). The median DA INT precision was higher than DA PYR precision (p < 0.001, U test), but not consistently different from IDA INT precision (p = 0.06; Figure 7M). Thus, error correction and improved precision occur at the PYR-to-INT interface in cortical networks with distinct architectures, the neocortex and CA1.

Finally, we compared error correction and precision improvement between regions. Error correction was observed along the PYR-to-INT interface in both neocortex and CA1, but did not differ consistently between pairs of connected units in the two regions (p = 0.09, U test; Figure S7A). The median precision of CA1 DA PYRs was 3.6 ms, poorer than the precision of the neocortical DA PYRs (3.2 ms; p < 0.001, U test; Figure S7E). Consistent with this, the median precision of IDA INT in CA1 (2.8 ms) was poorer than in the neocortex (2.3 ms; p < 0.001, U test; Figure S7F). When examined on a pair-by-pair basis, monosynaptic PYR-to-INT pairs exhibited higher improvement of precision in the neocortex (median 1.6 ms) compared with CA1 (0.8 ms; p = 0.03, U test; Figure S7B). Compared with the neocortex, the degree of convergence was higher in CA1, with a median [IQR] of 18 [10 23] presynaptic PYRs (n = 124 IDA INTs; p < 0.001, U test). Thus, the extent of error correction and improved precision may also depend on circuit architecture.

DISCUSSION

We found that, compared with DA PYRs, postsynaptic INT spike trains enabled better reconstruction of the WN signal and exhibited higher precision, particularly when more presynaptic PYRs were recruited. Error correction and improved precision were consistent with nonlinear amplification of coincident presynaptic inputs. Furthermore, postsynaptic INTs exhibited temporal coding, whereas DA INTs did not.

Signal transmission in the presence of noise

The present experiments were designed to measure the precision and reliability of spike generation and transmission in the presence of background inputs from thousands of terminals (Gulyás et al., 1999). Using WN signals, we found that spike generation exhibits a precision of 2–4 ms. However, even a 3 ms jitter poses a challenge to stable signal propagation. Some pathways include exceptionally strong synapses, enabling stable spike transmission at nearly a 1:1 ratio (Henze et al., 2002; Szegedi et al., 2017; Jouhanneau et al., 2018), but most cortico-cortical connections are weak. Indeed, an LL transmission model could not drive postsynaptic cells reliably. An alternative to LL transmission is the combination of multiple weak presynaptic inputs. Although expected to saturate when driven by correlated noise (Zohary et al., 1994), linear summation may transmit spikes efficiently (Reich et al., 2001). Indeed, increasing the presynaptic pool size experimentally induced higher precision. However, simple summation of the simultaneously recorded spike trains did not replicate the experimental findings. In contrast, nonlinear summation of coincidence spikes (Stuart and Häusser, 2001) was able to drive postsynaptic spiking more precisely than the driving trains. We found that error correction and precision improvement via CD of multiple weak synaptic inputs are particularly advantageous when failure of a synapse or a presynaptic neuron occurs. As a practical example of how error correction may work under natural conditions, the WN signal can be viewed as an analog of external input encoding specific sensory information to a pool of neurons, which also receives other activity. Then, every neuron will spike differently due to the background "noise," but some spikes of the neurons in the activated pool will occur sufficiently close in time to drive postsynaptic activity more robustly than any of the elements of the pool.

Temporal coding and coincidence detection

In contrast to intracellular current injection, we applied the WN signals to small groups of adjacent neurons using photocurrents. Current injection is used widely to probe spike generation (Asanuma et al., 1968; Mainen and Seinowski, 1995; Bacci and Huguenard, 2006; Cossell et al., 2015; Pala and Petersen, 2015), but is clearly a non-physiological intervention. We found that neuronal response properties are qualitatively different when neurons are driven directly via photocurrents, compared with synaptic inputs. The direct optogenetic activation is largely somatic, whereas excitatory synaptic activation is largely mediated via synapses on the dendrites. Dendrites of PV cells support nonlinear summation of coincident inputs (Hu et al., 2010; Chiovini et al., 2014). While not tested in the present work, PYR dendrites are equipped with machinery for CD (Stuart and Häusser, 2001; Polsky et al., 2004; Ferrarese et al., 2018). Thus, we hypothesize that PYR-to-PYR transmission will also result in temporal coding by the postsynaptic PYRs.

Methodological considerations

The question of whether neurons transmit information via rate or temporal codes has been discussed extensively (König et al., 1996; Salinas and Sejnowski, 2001; Cohen and Kohn, 2011), and much of the debate is rooted in definitions. The temporal/rate code differentiation is distinct from the mode by which a population of neurons encodes information: a population code can be based on rates, and a single neuron code can be temporal (Rieke et al., 1997). One approach is to analyze spike trains from the perspective of a downstream target. Here, we operationally defined temporal coding as a reliability profile that peaks at a timescale shorter than the maximal tested timescale, but in fact, we placed rate/temporal coding on a continuous scale. This approach, which does not depend on any reconstruction filter, is based on a spike distance metric that involves convolving spike

trains with kernels of gradually increasing temporal support (Schreiber et al., 2003). Clearly, various kernels and other distance metrics are plausible (Victor and Purpura, 1996; van Rossum, 2001). When the number of spikes is fixed, the reliability profile necessarily increases monotonically. However, in the presence of spikes due to nonspecific inputs, the reliability profile may exhibit a local peak.

A classic approach for quantifying the precision and reliability of spike generation is to apply a current pulse to the tested neuron and measure latency and jitter of the resultant spiking. The response to a pulse attenuates over time, explores only a subset of the response space, and is influenced by background spiking. Indeed, previous work showed that, while the first spike after a current pulse may exhibit millisecond-timescale precision, additional spikes are less precise (Mainen and Seinowski, 1995; Bacci and Huguenard, 2006; Pala and Petersen, 2015). To overcome these limitations, WN signals were employed *in vitro* to study spike transmission (Marmarelis and Naka, 1972). WN signals applied *in vitro* allow one to maintain millisecond-timescale precision over multiple generated spikes (Mainen and Seinowski, 1995). The present work applied optogenetic WN signals to specific cell types for studying the properties of spike transmission in the intact, freely moving animal. Indeed, the approach developed here can be employed for studying spike transmission between diverse types of neurons, in various brain regions (Klausberger and Somogyi, 2008; Tremblay et al., 2016).

Dependence on circuit architecture

To allow generalization beyond the well-mapped circuits of initial sensory processing, we studied local networks in associative brain regions, the parietal cortex and CA1. We found that some temporal properties of spike generation and transmission are qualitatively similar: DA PYRs exhibited a precision of a few milliseconds, whereas IDA INTs exhibited error correction and improved precision. However, we also observed some differences. First, decoding quality and precision were consistently higher in the neocortex than in CA1. Second, improved precision was consistently higher in the neocortex. Some differences may be due to methodological details (light power, number of activated PYRs), whereas others may be functional (diversity between PYRs, number of presynaptic PYRs). Regardless of the differences, postsynaptic error correction, improved precision, and temporal coding were observed in both brain regions examined, suggesting convergent synaptic transmission as a general mechanism for precise information propagation along the cortical PYR-to-INT interface.

Limitations of the study

There are several limitations that should be emphasized. First, determining connectivity from finite data is necessarily biased toward stronger connections. Strong connections favor the LL and SP models of transmission more than the CD model, in which the effect of as few as two presynaptic spikes is amplified by the nonlinearity. Second, the finding that postsynaptic INTs exhibit temporal coding rests upon the assumption that the cells tested during synaptic activation (IDA INTs) and the cells tested during direct optogenetic activation (DA INTs) are identical. We verified that properties do not differ at the population level, but more work is required to directly test the assumption by synaptic and optogenetic activation of the exact same INTs. Third, the properties of spike transmission between PYRs, which are of great interest, remain unknown. Specifically, the present experiments did not explore the precision and reliability timescale of spike transmission from a presynaptic assembly of PYRs to a postsynaptic target PYR. Based on the present results, we hypothesize that IDA INTs facilitate error correction and precision improvement during the transmission of information from one pool of PYRs to another.

STAR★METHODS

Detailed methods are provided in the online version of this paper and include the following:

- KEY RESOURCES TABLE
- RESOURCE AVAILABILITY
  - Lead contact
  - Materials availability
  - Data and code availability
- EXPERIMENTAL MODEL AND SUBJECT DETAILS
- METHOD DETAILS
  - Probes and surgery
  - Recording sessions and WN signals
  - Spike detection and sorting
  - Determining monosynaptic connectivity
  - Filters and cross-validated reconstruction
  - Precision
  - Reliability timescale
  - Point process simulation of spike trains
  - Data-driven simulation of synthetic INT
- QUANTIFICATION AND STATISTICAL ANALYSIS
  - Statistical analysis

SUPPLEMENTAL INFORMATION

Supplemental information can be found online at https://doi.org/10.1016/j.celrep.2022.111383.

ACKNOWLEDGMENTS

We thank Ortal Amber-Vitos, György Buzsáki, Shigeyoshi Fujisawa, Roni Gattengo, Shir Mandelboum, Moshe Parnas, Lisa Roux, Jackie Schiller, Shir Sivroni, and Alexander Tarnavsky-Eitan for constructive comments. This work was supported by the United States-Israel Binational Science Foundation (BSF; 2015577), by the European Research Council (679253), by the Israel Science Foundation (638/16), by the Israel Science Foundation FIRST Program (1871/17), by the Rosetrees Trust (A1576), and by the Canadian Institutes of Health Research (CIHR), the International Development Research Centre (IDRC), the Israel Science Foundation (ISF), and the Azrieli Foundation (2558/18).

AUTHOR CONTRIBUTIONS

A.L. and E.S. conceived and designed the experiments. A.L., L.S., H.E.S., S.S., and E.S. constructed optoelectronic probes and implanted animals. A.L., L.S., H.E.S., and S.S. carried out experiments. A.L. and E.S. analyzed data, constructed models, and wrote the manuscript with input from all authors.

DECLARATION OF INTERESTS

The authors declare no competing interests.

Received: March 8, 2022
Revised: May 26, 2022
Accepted: August 28, 2022
Published: September 20, 2022

REFERENCES

Abeles, M. (1991). Corticonics (Cambridge University Press).

Aljadeff, J., Lansdell, B.J., Fairhall, A.L., and Kleinfeld, D. (2016). Analysis of neuronal spike trains, deconstructed. Neuron *91*, 221–259. https://doi.org/10.1016/j.neuron.2016.05.039.

Asanuma, H., Stoney, S.D., Jr., and Abzug, C. (1968). Relationship between afferent input and motor outflow in cat motorsensory cortex. J. Neurophysiol. *31*, 670–681. https://doi.org/10.1152/jn.1968.31.5.670.

Azouz, R., and Gray, C.M. (2000). Dynamic spike threshold reveals a mechanism for synaptic coincidence detection in cortical neurons in vivo. Proc. Natl. Acad. Sci. USA *97*, 8110–8115. https://doi.org/10.1073/pnas.130200797.

Bacci, A., and Huguenard, J.R. (2006). Enhancement of spike-timing precision by autaptic transmission in neocortical inhibitory interneurons. Neuron *49*, 119–130. https://doi.org/10.1016/j.neuron.2005.12.014.

Barlow, H.B. (1972). Single units and sensation: a neuron doctrine for perceptual psychology? Perception *1*, 371–394. https://doi.org/10.1068/p010371.

Bhandawat, V., Olsen, S.R., Gouwens, N.W., Schlief, M.L., and Wilson, R.I. (2007). Sensory processing in the Drosophila antennal lobe increases reliability and separability of ensemble odor representations. Nat. Neurosci. *10*, 1474–1482. https://doi.org/10.1038/nn1976.

Bialek, W., Rieke, F., de Ruyter van Steveninck, R.R., and Warland, D. (1991). Reading a neural code. Science *252*, 1854–1857. https://doi.org/10.1126/science.2063199.

Billimoria, C.P., DiCaprio, R.A., Birmingham, J.T., Abbott, L.F., and Marder, E. (2006). Neuromodulation of spike-timing precision in sensory neurons. J. Neurosci. *26*, 5910–5919. https://doi.org/10.1523/JNEUROSCI.4659-05.2006.

Braitenberg, P.D.V., and Schüz, P.D.A. (1998). Cortex: Statistics and Geometry of Neuronal Connectivity (Springer-Verlag).

Bryant, H.L., and Segundo, J.P. (1976). Spike initiation by transmembrane current: a white-noise analysis. J. Physiol. *260*, 279–314. https://doi.org/10.1113/jphysiol.1976.sp011516.

Buračas, G.T., Zador, A.M., DeWeese, M.R., and Albright, T.D. (1998). Efficient discrimination of temporal patterns by motion-sensitive neurons in primate visual cortex. Neuron *20*, 959–969. https://doi.org/10.1016/s0896-6273(00)80477-8.

Cash, S., and Yuste, R. (1999). Linear summation of excitatory inputs by CA1 pyramidal neurons. Neuron *22*, 383–394. https://doi.org/10.1016/s0896-6273(00)81098-3.

Chiovini, B., Turi, G.F., Katona, G., Kaszás, A., Pálfi, D., Maák, P., Szalay, G., Szabó, M.F., Szabó, G., Szadai, Z., et al. (2014). Dendritic spikes induce ripples in parvalbumin interneurons during hippocampal sharp waves. Neuron *82*, 908–924. https://doi.org/10.1016/j.neuron.2014.04.004.

Cohen, M.R., and Kohn, A. (2011). Measuring and interpreting neuronal correlations. Nat. Neurosci. *14*, 811–819. https://doi.org/10.1038/nn.2842.

Cossell, L., Iacaruso, M.F., Muir, D.R., Houlton, R., Sader, E.N., Ko, H., Hofer, S.B., and Mrsic-Flogel, T.D. (2015). Functional organization of excitatory synaptic strength in primary visual cortex. Nature *518*, 399–403. https://doi.org/10.1038/nature14182.

Cover, T.M., and Thomas, J.A. (1991). Elements of Information Theory (John Wiley and Sons).

de Boer, R., and Kuyper, P. (1968). Triggered correlation. IEEE Trans. Biomed. Eng. *15*, 169–179. https://doi.org/10.1109/tbme.1968.4502561.

de Ruyter Van Steveninck, R., and Bialek, W. (1988). Real-time performance of a movement-sensitive neuron in the blowfly visual system: coding and information transfer in short spike sequences. Proc. R. Soc. Lond. Ser. B Biol. Sci. *234*, 379–414. https://doi.org/10.1098/rspb.1988.0055.

DiCarlo, J.J., Johnson, K.O., and Hsiao, S.S. (1998). Structure of receptive fields in area 3b of primary somatosensory cortex in the alert monkey. J. Neurosci. *18*, 2626–2645. https://doi.org/10.1523/JNEUROSCI.18-07-02626.1998.

Diesmann, M., Gewaltig, M.O., and Aertsen, A. (1999). Stable propagation of synchronous spiking in cortical neural networks. Nature *402*, 529–533. https://doi.org/10.1038/990101.

Eggermont, J.J., Johannesma, P.M., and Aertsen, A.M. (1983). Reverse-correlation methods in auditory research. Q. Rev. Biophys. *16*, 341–414. https://doi.org/10.1017/s0033583500005126.

Ferrarese, L., Jouhanneau, J.S., Remme, M.W.H., Kremkow, J., Katona, G., Rózsa, B., Schreiber, S., and Poulet, J.F.A. (2018). Dendrite-specific amplification of weak synaptic input during network activity in vivo. Cell Rep. *24*, 3455–3465.e5. https://doi.org/10.1016/j.celrep.2018.08.088.

Galarreta, M., and Hestrin, S. (2001). Spike transmission and synchrony detection in networks of GABAergic interneurons. Science *292*, 2295–2299. https://doi.org/10.1126/science.1061395.

Griffith, J.S. (1963). On the stability of brain-like structures. Biophys. J. *3*, 299–308. https://doi.org/10.1016/s0006-3495(63)86822-8.

Gulyás, A.I., Megías, M., Emri, Z., and Freund, T.F. (1999). Total number and ratio of excitatory and inhibitory synapses converging onto single interneurons of different types in the CA1 area of the rat hippocampus. J. Neurosci. *19*, 10082–10097. https://doi.org/10.1523/JNEUROSCI.19-22-10082.1999.

Henze, D.A., Wittner, L., and Buzsáki, G. (2002). Single granule cells reliably discharge targets in the hippocampal CA3 network in vivo. Nat. Neurosci. *5*, 790–795. https://doi.org/10.1038/nn887.

Hu, H., Martina, M., and Jonas, P. (2010). Dendritic mechanisms underlying rapid synaptic activation of fast-spiking hippocampal interneurons. Science *327*, 52–58. https://doi.org/10.1126/science.1177876.

Jeanne, J.M., and Wilson, R.I. (2015). Convergence, divergence, and reconvergence in a feedforward network improves neural speed and accuracy. Neuron *88*, 1014–1026. https://doi.org/10.1016/j.neuron.2015.10.018.

Joris, P.X., Carney, L.H., Smith, P.H., and Yin, T.C. (1994). Enhancement of neural synchronization in the anteroventral cochlear nucleus. I. Responses to tones at the characteristic frequency. J. Neurophysiol. *71*, 1022–1036. https://doi.org/10.1152/jn.1994.71.3.1022.

Jouhanneau, J.S., Kremkow, J., and Poulet, J.F.A. (2018). Single synaptic inputs drive high-precision action potentials in parvalbumin expressing GABA-ergic cortical neurons in vivo. Nat. Commun. *9*, 1540–1611. https://doi.org/10.1038/s41467-018-03995-2.

Kadir, S.N., Goodman, D.F.M., and Harris, K.D. (2014). High-dimensional cluster analysis with the masked EM algorithm. Neural Comput. *26*, 2379–2394. https://doi.org/10.1162/NECO_a_00661.

Kara, P., Reinagel, P., and Reid, R.C. (2000). Low response variability in simultaneously recorded retinal, thalamic, and cortical neurons. Neuron *27*, 635–646. https://doi.org/10.1016/s0896-6273(00)00072-6.

Klausberger, T., and Somogyi, P. (2008). Neuronal diversity and temporal dynamics: the unity of hippocampal circuit operations. Science *321*, 53–57. https://doi.org/10.1126/science.1149381.

König, P., Engel, A.K., and Singer, W. (1996). Integrator or coincidence detector? The role of the cortical neuron revisited. Trends Neurosci. *19*, 130–137. https://doi.org/10.1016/s0166-2236(96)80019-1.

Kuenzel, T., Borst, J.G.G., and van der Heijden, M. (2011). Factors controlling the input-output relationship of spherical bushy cells in the gerbil cochlear nucleus. J. Neurosci. *31*, 4260–4273. https://doi.org/10.1523/JNEUROSCI.5433-10.2011.

London, M., Roth, A., Beeren, L., Häusser, M., and Latham, P.E. (2010). Sensitivity to perturbations in vivo implies high noise and suggests rate coding in cortex. Nature *466*, 123–127. https://doi.org/10.1038/nature09086.

Mainen, Z.F., and Sejnowski, T.J. (1995). Reliability of spike timing in neocortical neurons. Science *268*, 1503–1506. https://doi.org/10.1126/science.7770778.

Marmarelis, P.Z., and Marmarelis, V.Z. (1978). Analysis of Physiological Systems: The White-Noise Approach (Plenum Press).

Marmarelis, P.Z., and Naka, K. (1972). White-noise analysis of a neuron chain: an application of the Wiener theory. Science *175*, 1276–1278. https://doi.org/10.1126/science.175.4027.1276.

Midorikawa, M., and Sakaba, T. (2015). Imaging exocytosis of single synaptic vesicles at a fast CNS presynaptic terminal. Neuron *88*, 492–498. https://doi.org/10.1016/j.neuron.2015.09.047.

Mitra, P.P., and Pesaran, B. (1999). Analysis of dynamic brain imaging data. Biophys. J. *76*, 691–708. https://doi.org/10.1016/S0006-3495(99)77236-X.

Noked, O., Levi, A., Someck, S., Amber-Vitos, O., and Stark, E. (2021). Bidirectional optogenetic control of inhibitory neurons in freely-moving mice. IEEE Trans. Biomed. Eng. *68*, 416–427. https://doi.org/10.1109/TBME.2020.3001242.

Pachitariu, M., Steinmetz, N.A., Kadir, S.N., Carandini, M., and Harris, K.D. (2016). Kilosort: realtime spike-sorting for extracellular electrophysiology with hundreds of channels. Preprint at bioXriv. https://doi.org/10.1101/061481.

Pala, A., and Petersen, C.C.H. (2015). In vivo measurement of cell-type-specific synaptic connectivity and synaptic transmission in layer 2/3 mouse barrel cortex. Neuron *85*, 68–75. https://doi.org/10.1016/j.neuron.2014.11.025.

Panzeri, S., and Treves, A. (1996). Analytical estimates of limited sampling biases in different information measures. Network *7*, 87–107. https://doi.org/10.1080/0954898X.1996.11978656.

Platkiewicz, J., Stark, E., and Amarasingham, A. (2017). Spike-centered jitter can mistake temporal structure. Neural Comput. *29*, 783–803. https://doi.org/10.1162/NECO_a_00927.

Polsky, A., Mel, B.W., and Schiller, J. (2004). Computational subunits in thin dendrites of pyramidal cells. Nat. Neurosci. *7*, 621–627. https://doi.org/10.1038/nn1253.

Pouille, F., and Scanziani, M. (2001). Enforcement of temporal fidelity in pyramidal cells by somatic feed-forward inhibition. Science *293*, 1159–1163. https://doi.org/10.1126/science.1060342.

Reich, D.S., Mechler, F., and Victor, J.D. (2001). Independent and redundant information in nearby cortical neurons. Science *294*, 2566–2568. https://doi.org/10.1126/science.1065839.

Reid, R.C., Victor, J.D., and Shapley, R.M. (1997). The use of m-sequences in the analysis of visual neurons: linear receptive field properties. Vis. Neurosci. *14*, 1015–1027. https://doi.org/10.1017/s0952523800011743.

Reyes, A.D. (2003). Synchrony-dependent propagation of firing rate in iteratively constructed networks in vitro. Nat. Neurosci. *6*, 593–599. https://doi.org/10.1038/nn1056.

Rieke, F., Warland, D., Steveninck, R., and Bialek, W. (1997). Spikes: Exploring the Neural Code (MIT Press).

Rossant, C., Leijon, S., Magnusson, A.K., and Brette, R. (2011). Sensitivity of noisy neurons to coincident inputs. J. Neurosci. *31*, 17193–17206. https://doi.org/10.1523/JNEUROSCI.2482-11.2011.

Rothman, J.S., Young, E.D., and Manis, P.B. (1993). Convergence of auditory nerve fibers onto bushy cells in the ventral cochlear nucleus: implications of a computational model. J. Neurophysiol. *70*, 2562–2583. https://doi.org/10.1152/jn.1993.70.6.2562.

Sadeh, S., and Clopath, C. (2021). Inhibitory stabilization and cortical computation. Nat. Rev. Neurosci. *22*, 21–37. https://doi.org/10.1038/s41583-020-00390-z.

Salinas, E., and Sejnowski, T.J. (2001). Correlated neuronal activity and the flow of neuronal information. Nat. Rev. Neurosci. *2*, 539–550. https://doi.org/10.1038/35086012.

Schreiber, S., Fellous, J.M., Whitmer, D., Tiesinga, P., and Sejnowski, T.J. (2003). A new correlation-based measure of spike timing reliability. Neurocomputing *52-54*, 925–931. https://doi.org/10.1016/S0925-2312(02)00838-X.

Sloin, Hadas, E, Bikovski, Lior, Levi, Amir, Amber-Vitos, Ortal, Katz, Tomer, Spivak, Lidor, Someck, Shirly, Gattegno, Roni, Sivroni, Shir, Sjulson, Lucas, and Stark, Eran (2022). Hybrid Offspring of C57BL/6J Mice Exhibit Improved Properties for Neurobehavioral Research. eNeuro *9* (*4*), 1–16. https://doi.org/10.1523/ENEURO.0221-22.2022.

Spivak, L., Levi, A., Sloin, H.E., Someck, S., and Stark, E. (2022). Deconvolution improves the detection and quantification of spike transmission gain from spike trains. Commun. Biol. *5*, 520. https://doi.org/10.1038/s42003-022-03450-5.

Stark, E., and Abeles, M. (2009). Unbiased estimation of precise temporal correlations between spike trains. J. Neurosci. Methods *179*, 90–100. https://doi.org/10.1016/j.jneumeth.2008.12.029.

Stark, E., Koos, T., and Buzsáki, G. (2012). Diode probes for spatiotemporal optical control of multiple neurons in freely moving animals. J. Neurophysiol. *108*, 349–363. https://doi.org/10.1152/jn.00153.2012.

Stark, E., Eichler, R., Roux, L., Fujisawa, S., Rotstein, H.G., and Buzsáki, G. (2013). Inhibition-induced theta resonance in cortical circuits. Neuron *80*, 1263–1276. https://doi.org/10.1016/j.neuron.2013.09.033.

Stark, E., Roux, L., Eichler, R., Senzai, Y., Royer, S., and Buzsáki, G. (2014). Pyramidal cell-interneuron interactions underlie hippocampal ripple oscillations. Neuron *83*, 467–480. https://doi.org/10.1016/j.neuron.2014.06.023.

Stuart, G.J., and Häusser, M. (2001). Dendritic coincidence detection of EPSPs and action potentials. Nat. Neurosci. *4*, 63–71. https://doi.org/10.1038/82910.

Swadlow, H.A., and Gusev, A.G. (2001). The impact of ‘‘bursting’’ thalamic impulses at a neocortical synapse. Nat. Neurosci. *4*, 402–408. https://doi.org/10.1038/86054.

Szegedi, V., Molnár, G., Paizs, M., Csakvari, E., Barzó, P., Tamás, G., and Lamsa, K. (2017). High-precision fast-spiking basket cell discharges during complex events in the human neocortex. eNeuro *4*, ENEURO.0260–17.2017. https://doi.org/10.1111/j.1460-9568.1991.tb00845.x.

Thomson, A.M., and Radpour, S. (1991). Excitatory connections between CA1 pyramidal cells revealed by spike triggered averaging in slices of rat hippocampus are partially NMDA receptor mediated. Eur. J. Neurosci. 3, 587–601.

Tran-Van-Minh, A., Abrahamsson, T., Cathala, L., and DiGregorio, D.A. (2016). Differential dendritic integration of synaptic potentials and calcium in cerebellar interneurons. Neuron *91*, 837–850. https://doi.org/10.1016/j.neuron.2016.07.029.

Tremblay, R., Lee, S., and Rudy, B. (2016). GABAergic Interneurons in the neocortex: from cellular properties to circuits. Neuron *91*, 260–292. https://doi.org/10.1016/j.neuron.2016.06.033.

van Rossum, M.C. (2001). A novel spike distance. Neural Comput. *13*, 751–763. https://doi.org/10.1162/089976601300014321.

Victor, J.D., and Purpura, K.P. (1996). Nature and precision of temporal coding in visual cortex: a metric-space analysis. J. Neurophysiol. *76*, 1310–1326. https://doi.org/10.1152/jn.1996.76.2.1310.

Xu-Friedman, M.A., and Regehr, W.G. (2005). Dynamic-clamp analysis of the effects of convergence on spike timing. I. Many synaptic inputs. J. Neurophysiol. *94*, 2512–2525. https://doi.org/10.1152/jn.01307.2004.

Yizhar, O., Fenno, L.E., Prigge, M., Schneider, F., Davidson, T.J., O'Shea, D.J., Sohal, V.S., Goshen, I., Finkelstein, J., Paz, J.T., et al. (2011). Neocortical excitation/inhibition balance in information processing and social dysfunction. Nature *477*, 171–178. https://doi.org/10.1038/nature10360.

Zohary, E., Shadlen, M.N., and Newsome, W.T. (1994). Correlated neuronal discharge rate and its implications for psychophysical performance. Nature *370*, 140–143. https://doi.org/10.1038/370140a0.

STAR★METHODS

KEY RESOURCES TABLE

REAGENT or RESOURCE	SOURCE	IDENTIFIER
Experimental models: Organisms/strains		
Mouse: Ai32	The Jackson Laboratory	RRID:IMSR_JAX:012569
Mouse: CaMKII-Cre	The Jackson Laboratory	RRID:IMSR_JAX:005359
Mouse: PV-Cre	The Jackson Laboratory	RRID:IMSR_JAX:008069
Mouse: FVB/NJ	The Jackson Laboratory	RRID:IMSR_JAX:001800
Bacterial and virus strains		
rAVV5/CamKII-hChR2(H134R)-mCherry	UNC	AV4317K
Software and algorithms		
MATLAB	Mathworks	http://www.mathworks.com
KlustaKwik3	Kadir et al. (2014)	https://github.com/kwikteam/klusta
Kilosort2	Pachitariu et al. (2016)	https://github.com/MouseLand/Kilosort
MATLAB code for spike timing analysis	This paper	https://doi.org/10.5281/zenodo.7025664
Other		
Silicon probe: four shanks, 32 channels	NeuroNexus	Buzaski32 (H32 21 mm)
Silicon probe: six shanks, 64 channels	Diagnostic Biochips	Stark64 (P64-7)
Silicon probe: two dual-sided shanks, 64 channels	Diagnostic Biochips	Dual-sided64 (P32-1-D)
Silicon probe: single shank, 32 channels	NeuroNexus	Edge32 linear (A1x32-Edge)
Intan RHD2000	Intan Technologies	http://intantech.com/products_RHD2000.html
Digital signal processor	Tucker-Davis Technologies	RX8
Precision current source	Stark et al. (2012)	N/A

RESOURCE AVAILABILITY

Lead contact

Further information and requests for resources should be directed to and will be fulfilled by the lead contact, Eran Stark (eranstark@tauex.tau.ac.il).

Materials availability

This study did not generate new unique reagents.

Data and code availability

- All data reported in this paper will be shared by the lead contact upon request.
- All original code has been deposited at Zenodo and is publicly available as of the date of publication. DOIs are listed in the key resources table.
- Any additional information required to reanalyze the data reported in this paper is available from the lead contact upon request.

EXPERIMENTAL MODEL AND SUBJECT DETAILS

14 freely moving C57BL/NJ or hybrid mice (13 males, one female) were used in this study: 12 animals for experiments in neocortex, and nine mice for CA1 recordings (seven of the mice were used in both areas; Table S1). Mice aged 16 weeks (median; IQR: [12 20]) at the time of implantation. Animals were healthy and weighed 30 [24.2 31.4] g before implantation. Mice were single-housed to prevent damage to the implanted apparatus. Seven mice expressed ChR2 in PYR under the CaMKII promoter (CaMKII::ChR2). In five of these mice, ChR2 expression was achieved by crossing CaMKII-Cre males (JAX #005359, The Jackson Laboratory) with Ai32 females (#012569); in two other mice, viral vector injection was employed (CaMKII-hChR2-mCherry). Previous work showed that in these lines, there is no leakage of ChR2 to PV cells (Stark et al., 2013, 2014). The other seven mice expressed ChR2 in PV cells, generated by crossing PV-Cre males (#008069) with Ai32 females (PV::ChR2). All animal handling procedures were in accordance with Directive

2010/63/EU of the European Parliament, complied with Israeli Animal Welfare Law (1994), and approved by the Tel Aviv University Institutional Animal Care and Use Committee (IACUC #01-16-051).

METHOD DETAILS

Probes and surgery

All animals were implanted with high-density silicon probes attached to a movable microdrive and coupled with optical fibers. The probes used were: Stark64 (Diagnostic Biochips; six animals), Buzaski32 (NeuroNexus; five mice), Edge32 linear probe (NeuroNexus; one mouse), and Dual-sided64 (Diagnostic Biochips; two mice). The Stark64 probe consists of six shanks, spaced horizontally 200 μm apart, with each shank consisting of 10–11 recording sites, spaced vertically 15 μm apart. The Buzaski32 probe consists of four shanks, spaced horizontally 200 μm apart, with each shank consisting of eight recording sites, spaced vertically 20 μm apart. The Edge32 linear probe has a single shank consisting of 32 recording sites, spaced 20 μm apart. The Dual-sided64 probe consists of two dual-sided shanks, spaced horizontally 250 μm apart, with each shank consisting of 16 channels on each side (front and back), spaced vertically 20 μm apart. In each multi-shank probe, two to six shanks were equipped with an optical fiber, terminating about 50 μm above the top recording site. The linear probe was coupled to a single optical fiber. At the other end, fibers were coupled to diodes: 470 nm LEDs (LB P4SG, Osram), 367 nm LEDs (VLMU1610-365-135, Vishay), 450 nm laser diodes (PL450B, Osram), and 638 nm laser diodes (HL63603TG, Ushio) were used. Only blue (450 nm LDs and 470 nm LEDs) light sources were used in the reported experiments. The maximal driving current used was 50 mA, resulting in light power of 39 [29.9 42.8] μW measured at the tip of the probe (median [IQR] over n = 52 blue light sources).

Four of the CaMKII::ChR2 mice were hybrids (Sloin et al., 2022). Two mice (mA234 and mC41) were transgenic and hybrid, generated by crossing a CaMKII::ChR2 male (offspring of a CaMKII-Cre male and a Ai32 female) with an FVB/NJ female (JAX #001800). The other two (mF84, mF93) were hybrids, offspring of a C57BL/6J male (#000664) and an FVB/NJ female. The latter two mice were injected with a viral vector, rAVV5/CaMKII-hChR2(H134R)-mCherry (viral titer estimated at 5.6×10^{12} IU/mL; University of North Carolina viral core facility; courtesy of K. Deisseroth) to express ChR2 in PYR. The viral vector was injected stereotactically (Kopf) into parietal cortex and hippocampus at 8 different depths (AP -1.6, ML 1.1, DV 0.4 to 1.8 at 0.2 mm increments; 50 nL/site; Nanoject III, Drummond).

In all mice, probes were implanted in the neocortex above the hippocampus (AP/LM, −1.6/1.1 mm) under isoflurane (1%) anesthesia following previously-described procedures (Stark et al., 2012; Noked et al., 2021). Recordings were carried out five days a week. After every recording session, the probe was translated vertically downwards by up to 70 μm. Analyses of hippocampal regions included only recordings from the CA1 layer, recognized by the appearance of multiple high-amplitude units and iso-potential spontaneous ripple events.

Recording sessions and WN signals

In the beginning of each session, neuronal activity was inspected for spontaneous spiking activity, and if encountered, a baseline period of at least 15 min was recorded while the animal was in the home cage. The baseline period was followed by response mapping, using 50 ms light pulses at multiple power levels, used to determine for each light source a minimal light power P_{th} needed to generate spikes (“spiking threshold”). We denote the minimal light power that yields a population response (e.g., induced oscillations; Stark et al., 2014) as the “population threshold”. Next, WN signals were applied in the regime between the spiking threshold and the population threshold (typically, at three power levels: $P_{th}/2$, P_{th}, and $2P_{th}$). Signals were applied using every available light source, during spontaneous behavior in the home cage. In CaMKII::ChR2 mice, the median [IQR] light power used 5.5 [3.3 15] μW in neocortex (n = 139) and 7.8 [4.3 10] μW in CA1 (n = 113); in PV::ChR2, the power was 19 [12 20] μW (n = 49) in neocortex and 34 [20 34] μW (n = 21) in CA1. For every light source, 74 [41 100] WN repetitions (median [IQR]) were applied at each power level. The same was repeated for simultaneous activation of multiple light sources. Trials were carried out in a pseudorandom order between the different light sources and power levels. Each recording session ended with another baseline period of at least 15 min. Animals were equipped with a 3-axis accelerometer (ADXL-335, Analog Devices) for monitoring head movements.

The order and power levels of the WN signals were generated in MATLAB and routed to a digital signal processor (DSP; RX8, Tucker-Davis Technologies). The DSP issued a voltage command to a 16-channel linear current source (Stark et al., 2012) that drove the head-mounted diodes. For driving LDs, which have a nonlinear current-to-power transfer function, the power (P) was measured at multiple driving currents (I) to create a P-I lookup table. The table was used to translate the desired power output into a corresponding voltage, given as a command to the linear current source. The instantaneous light power to be administered by the system was calculated by multiplying the chosen power level by a filtered WN signal scaled between zero and one. The WN signal x(t) was generated once, by convolving uncorrelated Gaussian zero-mean WN sampled at 6 kHz with an alpha function ($t \cdot \exp(-t/\tau)$; $\tau = 3$ ms). The same 1 s long signal was used in all experiments. The actual light power varied, and was $P(t) = \mu + x(t)\sigma$, where σ was typically set to either $P_{th}/2$, P_{th}, or $2P_{th}$, and μ was set such that non-positive values were excluded.

Spike detection and sorting

Neural activity was filtered, amplified, multiplexed, and digitized on the headstage (0.1–7,500 Hz; x192, 16 bits, 20 KHz; RHD2132 or RHD2164, Intan Technologies), and recorded by an RHD2000 evaluation board (Intan Technologies). Offline, spikes were detected

and sorted into single units automatically using either KlustaKwik3 (Kadir et al., 2014) for probes with up to 11 sites/shank, or KiloSort2 (Pachitariu et al., 2016) for 16-site shanks. Automatic spike sorting was followed by manual adjustment of the clusters. Only well-isolated units were used for further analyses (Stark et al., 2013) (amplitude >40 μV; L-ratio <0.05; ISI index <0.2). Units were classified into putative PYR or PV-like INT using a Gaussian mixture model (Stark et al., 2013). In PV::ChR2 mice, units that exhibited a consistent ($p < 0.001$, Poisson test) increase in spiking rate during 50 ms DC pulses were tagged as PV cells. A total of 8,176 units (6,616 PYR and 1,560 INT) were recorded from neocortex (2,625 PYR, 843 INT) and CA1 (3,991 PYR, 717 INT) of 14 freely-moving mice during 162 sessions.

Units were maintained for WN analyses only if three criteria were fulfilled. (1) The unit was tested during at least five WN trials, emitted an average of at least two spikes per trial, and exhibited a DC response (3,998/6,784 (59%); excluding PYR recorded in PV::ChR2 mice; Figure S2A). Neither precision nor reliability is necessarily correlated with WN firing rate (Figure 1K). A DC response was defined as a DC gain consistently above 1 (Poisson test). The DC gain was in turn defined as the mean firing rate during WN trials, divided by the mean spontaneous firing rate (computed in the lack of any stimulation during non-theta immobility). (2) The unit exhibited an AC response, defined as consistent decoding quality (see below; permutation test). Of the units with a DC response, 1,617/3,998 (40%) units exhibited an AC response (Figure S2B). (3) The unit was reliable, defined as consistent reconstruction-based reliability (see below; permutation test). Of the units with an AC response, 662/1,617 (41%) units exhibited reliable spike trains (Figure S2C). Thus, after omitting units which did not fulfill all three criteria, the dataset consisted of 352 PYR and 310 INT (neocortex: 239 PYR and 165 INT; CA1: 113 PYR and 145 INT).

Determining monosynaptic connectivity

To determine whether a monosynaptic connection may exist between DA PYR and IDA INT, we constructed count cross-correlation histograms (CCHs; 0.4 ms bins) for putative pre- and postsynaptic spike train pairs during periods without WN. We defined the "spike transmission curve" as the impulse response of spike transmission between the pre- and postsynaptic neurons (Figure 3D). The spike transmission curve was estimated by the difference between the deconvolved CCH and the baseline, determined by median filtering of the count CCH and scaling to spikes/s (dividing by the number of presynaptic spikes and bin size; Spivak et al., 2022). "Spike transmission gain" (a measure of the synaptic efficacy; Swadlow and Gusev, 2001) was then defined as the area under the peak in the monosynaptic temporal region of interest (ROI; $0 < t \leq 5$ ms), extended until the causal zero-crossing points. Units that participated as a reference in a CCH that exhibited a consistent peak ($p < 0.001$, Bonferroni-corrected Poisson test on the count CCH compared to baseline; Stark and Abeles, 2009) in the monosynaptic ROI were defined as presynaptic excitatory cells. For every INT, connectivity probability (Figure 3A, right) was defined as the number of presynaptic PYRs, divided by the total number of simultaneously-recorded PYRs within a 600 μm range.

Filters and cross-validated reconstruction

For every unit, the spikes in a given dataset were used to construct a model (Rieke et al., 1997) (Figure 1A). The method used throughout the paper is an acausal modified Wiener filter, in which the spike triggered average (STA) filter is whitened by the autocorrelation of the output (i.e., the spike trains; see below). To determine whether results depend on the specific filter model, we also used previously described methods (STA and Wiener filters; causal and acausal; Aljadeff et al., 2016). Overall, twelve types of models were used (Figures S1A–S1D). The simplest model was a causal STA filter (De Boer and Kuyper, 1968). Denote the WN analog signal as x(t) and the unit spike times as T_i, i = 1..N. To generate the STA filter, the WN input was first standardized (zero mean, unit variance) to z(t). An a-causal STA vector $\vec{S}$ was generated by taking the average of segments of z(t) around each spike, $\vec{S} = \frac{1}{N}\sum_i \overrightarrow{Z(t_i)}$, where $t_i \in [\,T_i-50, T_i+50\,]$ ms. To generate a causal STA filter, we set the values at positive time lags to zero.

To account for the possibility that distinct signal features yield distinct two-spike sequences, we used conditional STA ("CSTA") filters, akin to the response-conditional ensembles approach (de Ruyter Van Steveninck and Bialek, 1988). Here, we first computed the inter-spike interval (ISI) preceding every spike; then, we partitioned the ISIs into ten equally-populated bins, and tagged every spike according to the corresponding bin. A distinct STA filter was then generated for every ISI bin, yielding a filter bank. Both causal and acausal CSTA filters were employed.

The WN signal employed was generated by filtering Gaussian WN of a finite duration with an alpha function, and therefore the spectrum of the signal actually applied to the brain was not white. While none of the analysis steps relied on the assumption that the signal is Gaussian or white, we considered a whitened version of the STA filter, equivalent to a first-order Wiener kernel (Marmarelis and Marmarelis, 1978). To generate the "Wiener" filter, the input signal was whitened before computing the STA. Whitening was done by multiplying the signal by the pseudo square root inverse of the signal covariance matrix (Aljadeff et al., 2016). To prevent amplification of high-frequency noise, the pseudoinverse matrix included only eigenvectors corresponding to the eigenvalues that together accounted for at least 90% of the signal variance. Finally, we considered a modified Wiener filter in which the STA filter was whitened by the output (i.e., the spike trains) autocorrelation. Here, the spike train ACH, specifically during the WN signal, was calculated for the same time range, and the ACH vector was transformed into a symmetric (Toeplitz) matrix, $\overleftrightarrow{A}$. The modified Wiener filter $\vec{w}$ is the deconvolution of the ACH from the STA, $\vec{w} = \overleftrightarrow{A}^{-1}\vec{s}$. For both the Wiener and the modified Wiener filters, "simple" and "conditional" models were employed.

All filters were computed using 10-fold cross-validation. First, available trials were divided into a training set (90% of the trials) and a test set (the rest). Second, a filter model was computed based on the training set, and used to reconstruct the WN input according to the spike trains of the test set trials (Figure 1A, bottom). Specifically, the WN pattern was reconstructed by convolving each test set spike train with the training set filter (for conditional filters, every spike was assigned an ISI bin and convolved with the corresponding filter in the bank). The procedure was repeated 10 times, so all trials were used for cross-validated reconstructions.

Single-trial decoding quality, q_i, was determined by the rank correlation coefficient (cc) between the standardized WN input, z, and the ith trial reconstruction, $\hat{z}_i$: $q_i = \rho(z, \hat{z}_i)$ (Figure 1B, top). Since Wiener filters were based on whitened signals, the correspondence of the signal actually applied to the brain with the Wiener-based reconstruction is necessarily penalized. Therefore, we also considered a pre-whitened STA filter ("PWSTA"), in which the filter itself is the same as the Wiener filter, but the signal against which the reconstruction was gauged, z, was whitened (note that the dually-white setting is also penalized). Statistical significance of the reconstruction was determined by random shuffling of the test set spike times within every trial, repeating reconstruction using the training set model, and computing an empirical p value for every trial. The overall decoding quality Q was defined as $Q = \frac{1}{N}\sum_i q_i$, the mean over all cross-validated single-trial values, and the p value was the geometric mean over all cross-validated single-trial p values. As an independent metric of reconstruction quality, we considered the mutual information (MI) between the input signal z and the reconstruction $\hat{z}_i$ (Rieke et al., 1997). Here, we evaluated the MI using a direct method, binning the two-dimensional distribution and correcting for bias using the Bayesian counting procedure (Panzeri and Treves, 1996). Finally, we considered the spectral coherence between the two signals. Coherence was derived from the cross-spectral density, estimated using multi-taper spectral methods (Mitra and Pesaran, 1999). Here, we used 5 discrete prolate spheroidal sequences, FFT of 512 points, and 50% overlap between adjacent windows.

Error correction and improved precision were observed in the neocortical PYR-to-INT interface for all twelve filter models employed (Figures S1E–S1I). This observation is consistent with the fact that all models rely on the STA, which by itself allows accurate reconstruction of arbitrary input (Bialek et al., 1991).

Precision

Only units that showed consistent decoding quality ($p < 0.05$) were subjected to precision quantification. Precision P was defined as the smallest temporal jitter that resulted in consistent deterioration of the cross-validated decoding quality Q (Figure 1B). All spikes were jittered using interval jitter (Platkiewicz et al., 2017) at half-window durations δ log-spaced between 0.4 ms and 102.4 ms, and Q was recalculated for each δ. The domain confined between the smallest δ that resulted in consistent deterioration (Wilcoxon test) of the cross-validated decoding quality and the next δ was used for a focused sweep. That domain was divided into 10 new δ values, spike trains were jittered at each δ, and Q calculated. Finally, precision was defined as the smallest focused window that resulted in consistent deterioration of the cross-validated decoding quality.

Reliability timescale

Reliability was defined as the cc between pairs of reconstructed trials, averaged over all trial pairs. In each trial, the signal was reconstructed using the cross-validated filter model. Consistent reliability was defined if the cc (averaged over all possible pairs of trials) was consistently larger than zero, tested using Wilcoxon's test. For each unit with consistent decoding quality and reliability, a reliability profile was calculated (Figure 1C). Every point on the reliability profile was defined as the mean cc between trial pairs $(\hat{x}_i, \hat{x}_j)$, where trials were "reconstructed" by filtering the spike trains with a Gaussian kernel with an SD of σ (Schreiber et al., 2003). The reliability profile was calculated for distinct σ values, log spaced between 0.4 and 102.4 ms. If the reliability profile peaked at σ other than the highest tested value, a focused sweep was used. The domain confined between the profile peak and the next σ was divided into 10 new values, and reliability was calculated for every σ. Reliability timescale Rp was defined as the σ which yielded the global maximum of the reliability profile.

Recorded neurons were divided into three groups based on their reliability profile. The first group, denoted as "rate coders", had monotonically increasing reliability profiles. The second group, denoted as "temporal coders", had a local maximum in the reliability profile at a timescale other than the highest tested σ. Temporal coders either had a reliability timescale at the local maximum (i.e., the local maximum was Rp), or had a second maximum at the highest tested σ (i.e., reliability timescale Rp differed from the local maximum). All other units were denoted as "undefined".

Point process simulation of spike trains

To test the framework of dissociating between temporal precision and reliability timescale (Figures 1D–1K), a WN point process simulation was conducted. Simulations proceeded with sampling frequency of $F_s = 2500$ Hz and N = 25 trials, each lasting T = 1 s. First, a firing rate profile f(t) was generated based on the WN signal, using a simplified STA-like kernel (a single sinusoid cycle with a frequency of 100 Hz). The kernel was treated as a template, which was convolved with the WN signal to create the firing rate profile. Second, given a rate function f(t), a "mother" signal spike train was generated as a non-homogeneous Poisson process by deciding at each time step whether a spike did or did not occur. This was done by random sampling from a binomial distribution with parameters $B(n,p)=(1,f(t)/F_s)$, followed by imposing a minimum inter-spike interval (ISI; 1–5 ms). Third, the mother signal spike train was replicated to generate N "daughter" signal spike trains, and each daughter spike train was modified as follows. (1) Each spike was

randomly jittered within a Gaussian window with a predetermined SD (“signal jitter”). (2) Up to N_{remove} spikes were randomly removed from each daughter train. (3) Up to $N_{add} = N_{remove}$ spikes were randomly added to the daughter spike train. Fourth, background (“noise”) spike trains were generated using a homogeneous Poisson process. The “mother” noise train was replicated to yield N daughter noise trains, and each was modified in the same manner as the daughter signal trains (using “noise jitter”, adding, and removing spikes). Each final spike train was a combination of a daughter signal spike train and a daughter noise spike train. Simulated spike trains were analyzed exactly as the experimentally recorded trains.

Parameter values used in artificial examples were as follows. Figure 1F: signal firing rate, $\lambda_1 = 25$ spikes/s; signal jitter, $\sigma_1 = 3$ ms; noise firing rate, $\lambda_2 = 0$ spikes/s; noise jitter, $\sigma_2 = 0$ ms; maximal number of added/removed spikes, $N_{add} = 25$. Figure 1G: $\lambda_1 = 100$ spikes/s; $\sigma_1 = 10$ ms; $\lambda_2 = 0$ spikes/s; $\sigma_2 = 0$ ms; $N_{add} = 0$. Figure 1H: $\lambda_1 = 100$ spikes/s; $\sigma_1 = 1$ ms; $\lambda_2 = 0$ spikes/s; $\sigma_2 = 0$ ms; $N_{add} = 10$. Figure 1I: $\lambda_1 = 100$ spikes/s; $\sigma_1 = 10$ ms; $\lambda_2 = 200$ spikes/s; $\sigma_2 = 60$ ms; $N_{add} = 0$.

Data-driven simulation of synthetic INT

To test the three transmission models (labeled line [LL], summed population [SP], and coincidence detection [CD]; Figure 4), synthetic INT (sINT) spike trains were stochastically generated from the real spike trains recorded from the DA PYR. For each IDA INT with presynaptic DA PYR, an sINT was generated. For the LL model (Figures S4A–S4D), the process of generating an sINT was as follows. First, the spike transmission curves and gains for all DA PYR and IDA INT pairs that exhibited putative monosynaptic connections were calculated (Figures 4B and 4C). For a single pair, the spike trains of the DA PYR observed during WN stimulation were convolved with the transmission curve of that specific pair to generate a rate function (Figure 4D). Therefore, for every WN trial, a different rate function was generated based on the DA PYR spike train actually observed. Since the WN signals were applied at multiple power levels using different combinations of light sources, a single experimental setup from which the actual DA PYR spike trains were extracted was selected. The selected experimental setup was that which yielded the highest DC gain for the postsynaptic IDA INT. From each rate function, a synthetic spike train (ST_1, with mean firing rate FR_1) was generated stochastically. A second spike train (ST_2), representing background activity (omitted for the “noiseless” condition; Figure 4E), was generated randomly as a homogeneous Poisson process with mean firing rate FR_2. Denoting the firing rate of the IDA INT during WN stimulation by FR_{WN}, the sINT spike trains were generated such that if $FR_1 \geq FR_{WN}$, no background activity was added. Otherwise, background activity was added such that $FR_1 + FR_2 = FR_{WN}$.

For the SP model (Figures S4E–S4I), the same procedure was followed, with the exception that a joint ST_1 was generated by linear summation of all presynaptic rate functions.

For the CD model (Figures S4J–S4T), two different implementations were used to generate the rate functions and the synthetic spike trains (ST_1). The first (CD_1) was a synaptic multiplication model in which the CD rate function is y(t) at time t was defined, for n(t) > 1, as

$$y(t) = \prod_{i=1}^{n(t)} (x_i(t) + \gamma) - \gamma^{n(t)},$$

where n(t) is the number of simultaneously-activate synapses at time t, $x_i(t)$ is the input rate function from the i'th DA PYR, and γ is a scalar non-negative non-linearity factor. When n(t) = 1, $y(t) = x_i(t)$; and when n(t) = 0, y(t) = 0. The second implementation (CD_2) was a nonlinear summation model. For CD_2, the rate function is y(t) = 0 when n(t) = 0; for $n(t) \geq 1$,

$$y(t) = \sum_{i=1}^{n(t)} x_i(t)\gamma^{n(t)-1}.$$

Thus, in both implementations, isolated spikes were not amplified, whereas coincident spiking yielded supra-linear amplification. The fact that only a subset of the PYR activated by the optogenetic input can be recorded limits the performance of any data-driven model. Thus, the models are oblivious to all unrecorded DA PYR that are presynaptic to the IDA INT. CD sINT precision is higher (Figure 4H) and more similar to IDA INT precision (Figure 4I) when more presynaptic PYRs are recorded.

QUANTIFICATION AND STATISTICAL ANALYSIS

Statistical analysis

In all statistical tests a significance threshold of $\alpha = 0.05$ was used. An exception was the threshold used for determining whether two units exhibit monosynaptic connectivity ($\alpha = 0.001$). In all cases, non-parametric testing was used. All statistical details (n, median, IQR) can be found in the main text and figure legends. To estimate whether fractions are larger or smaller than expected by chance, an exact one-tailed Binomial test was used. Differences in the proportions of observations of two categorical variables were tested with a *G*-test. Differences between two group medians were tested with either Mann-Whitney's one-tailed *U*-test (unpaired samples) or Wilcoxon's paired signed-rank one-tailed test. Association between parameters was quantified using Spearman's rank correlation and tested with a permutation test. For all figures, ns, p > 0.05; *, p < 0.05; **, p < 0.01; ***, p < 0.001.

Cell Reports, Volume *40*

Supplemental information

Error correction and improved precision of spike timing in converging cortical networks

Amir Levi, Lidor Spivak, Hadas E. Sloin, Shirly Someck, and Eran Stark

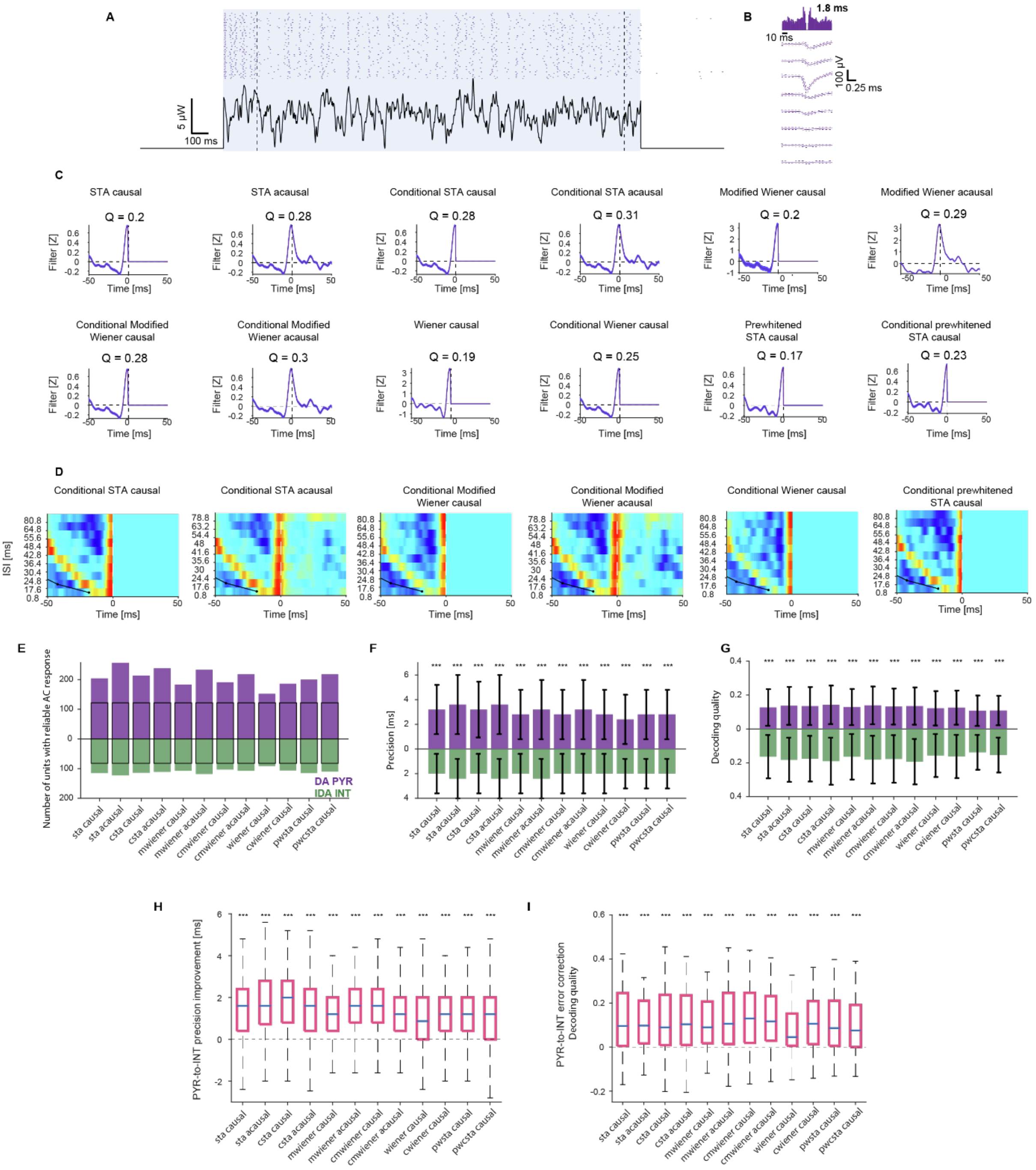

Figure S1. Error correction and improved precision are observed for all twelve filter models employed. Related to Figure 1.

(**A**) Spike trains of an example neocortical DA PYR (same as in **Figure 2C**) during 50 (out of 200) WN trials. (**B**) ACH and waveform of the DA PYR in **A**. (**C**) Twelve models for cross-validated reconstruction. For each model, a filter is generated, based on the WN signal and the spike trains. Decoding quality for all trials is denoted. When conditional models are used, the panel shows the unconditioned filter (not used for reconstruction). (**D**) For conditional models, a filter bank is generated, consisting of one filter for each ISI range. (**E**) The acausal STA filter yields the largest number of AC responsive neocortical DA PYR (257; *purple*) and IDA INT (123; *green*), whereas the causal Wiener filter yields the smallest numbers (152 DA PYR, 591 IDA INT). Black rectangles show the number of AC responsive units common to all filter models (122 DA PYR, 82 IDA INT). (**F**) At the population level, IDA INT exhibit higher precision than DA PYR, regardless of filter model employed. Here and in **G**, */**/***: p<0.05/p<0.01/p<0.001; *U*-test. (**G**) At the population level, IDA INT exhibit higher decoding quality than DA PYR, regardless of filter model. (**H-I**) For connected pairs, precision improvement (**H**) and error correction (**I**) are observed for all filter models. ***: p<0.001, Wilcoxon test.

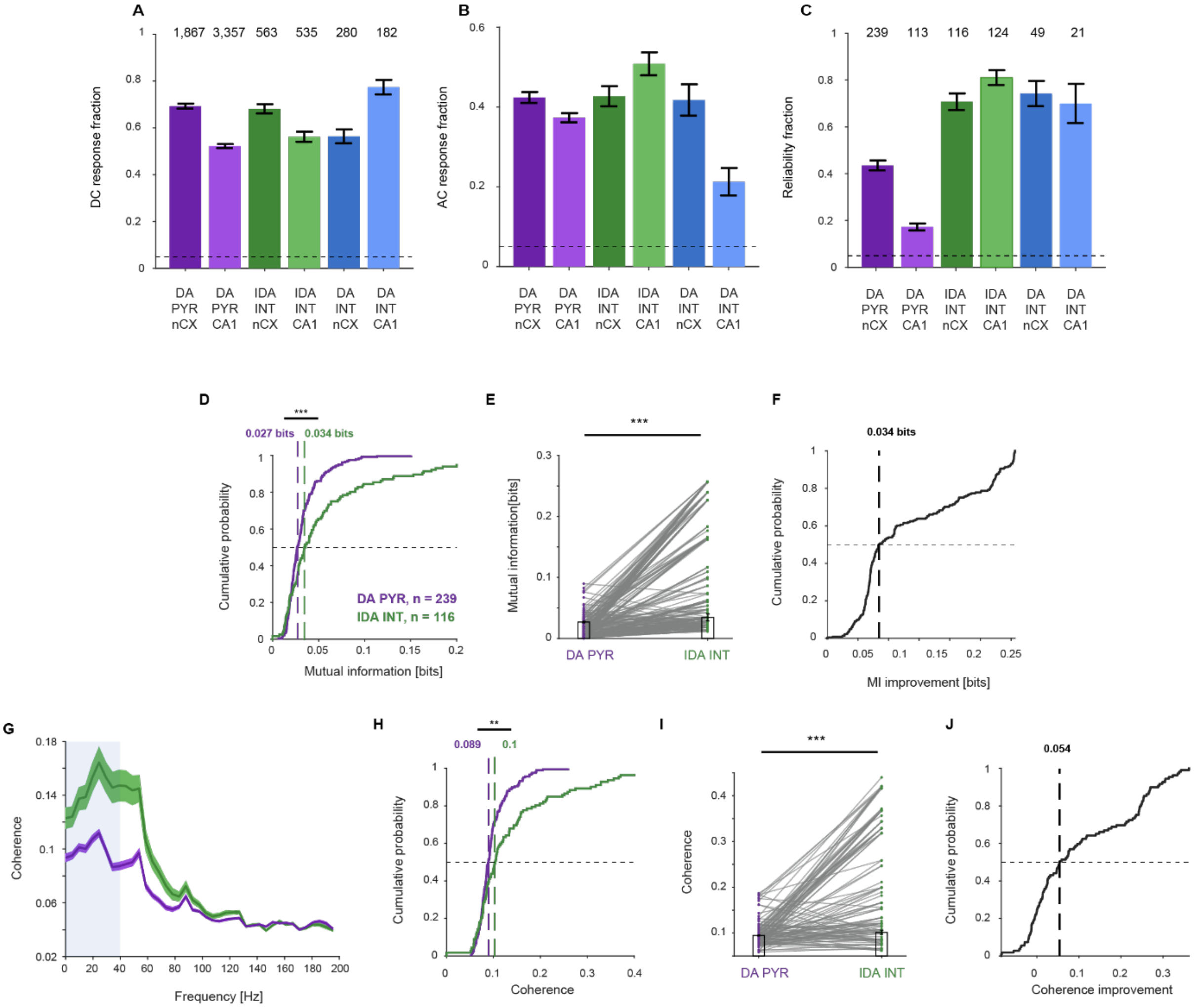

Figure S2. Error correction among the PYR-to-INT interface is evident when quantified using mutual information or spectral coherence. Related to Figure 2.

(**A-C**) Optogenetic WN experiments have low yield. The six categories of units analysed include DA PYR, IDA INT and DA INT, in the neocortex (nCX) and in hippocampal CA1. PYR recorded in PV::ChR2 mice were omitted from the data base. (**A**) Numbers above each bar denote the total number of recorded units of each category. Fraction of units with a "DC response" out of all units recorded during WN experiments. Here and in **B**-**C**, error bars indicate SEM, and horizontal dashed lines indicate chance level (0.05). Of the tested units, 3,998/6,784 (59%) exhibited a DC response. (**B**) Fraction of units with an AC response out of the units with a DC response. Of the units with a DC response, 1,617/3,998 (40%) units exhibited an AC response. (**C**) Fraction of reliable units out of all units with an AC response. Of the units with an AC response, 662/1,617 (41%) units exhibited reliable spike trains. Overall, 662/6,784 (9.8%) of the units recorded during WN experiments exhibited reliable spike trains and were used in this work; the precise numbers for each category are denoted above every bar.

(**D-F**) Mutual information (MI) is higher for IDA INT, compared to DA PYR. (**D**) As a population, IDA INT exhibit higher MI than DA PYR. Here and in **E**, vertical dashed lines indicate median values; **/***: $p<0.01/p<0.001$, *U*-test. (**E**) In 199/229 (87%) connected pairs, the MI for IDA INT is higher than for presynaptic DA PYR. Here and in **F**, ***: $p<0.001$, Wilcoxon test. (**F**) MI improvement (median, 0.034 bits) between connected DA PYR/IDA INT pairs.

(**G-J**) Spectral coherence magnitude is higher for IDA INT, compared to DA PYR. (**G**) Averaged coherence profiles of the two populations (mean±SEM). (**H**) As a population, IDA INT exhibit higher coherence (averaged over the 0-40 Hz band; see light blue rectangle in **D**) than DA PYR. (**I**) In 109/143 (76%) connected pairs, the coherence of IDA INT is higher than for presynaptic DA PYR. (**J**) Coherence improvement (median, 0.054) between connected DA PYR/IDA INT pairs.

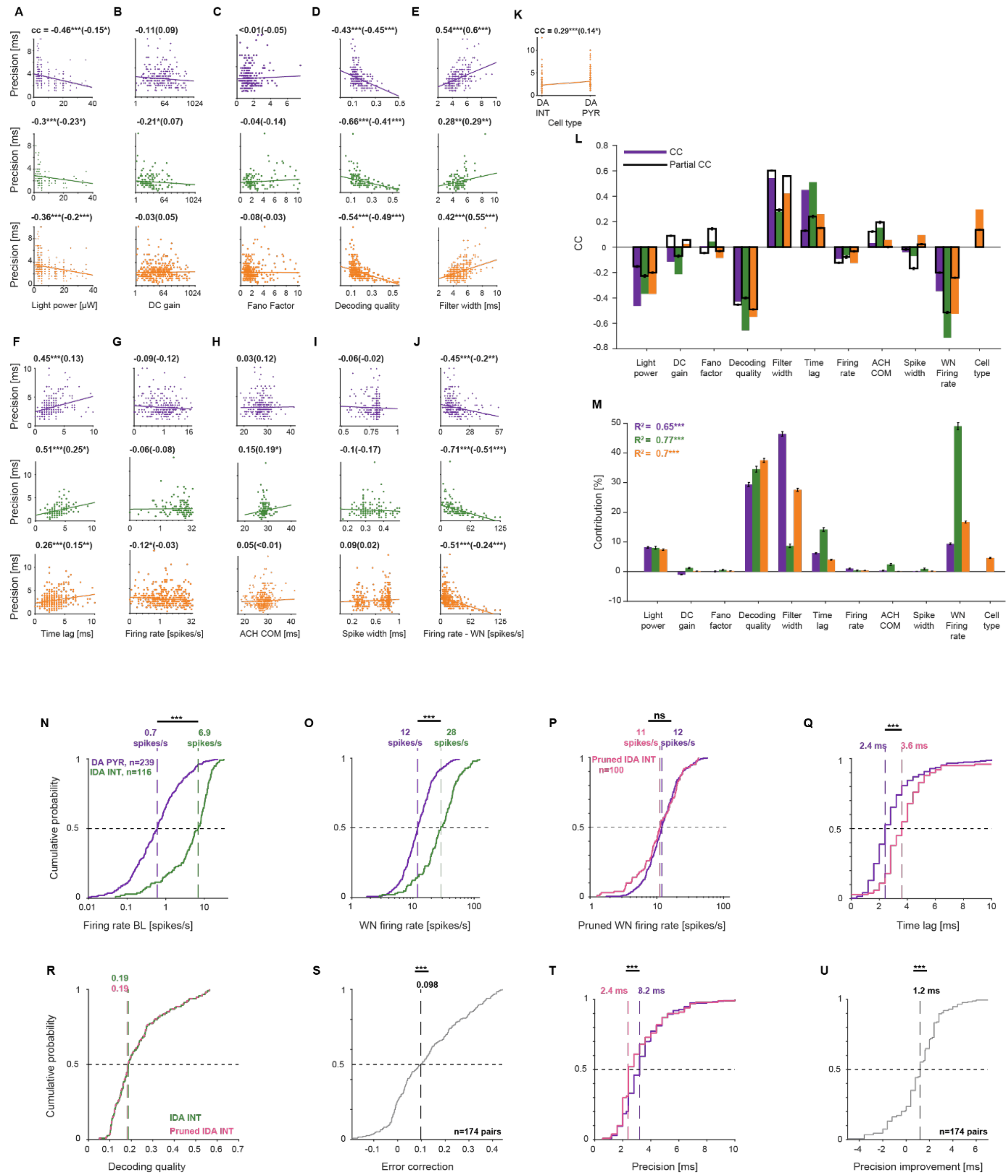

Figure S3. Precision of DA PYR and IDA INT depends mainly on filter width and WN firing rate, but IDA INT precision is preserved when IDA INT WN firing rates are equated to DA PYR WN rates. Related to Figure 2.

(**A-M**) Precision depends mainly o filter width and WN firing rate. (**A**) Scatter plot of the precision and the mean light power of the WN signal (*top*, DA PYR; *middle*, IDA INT; *bottom*, both). Here and in **B-K**, numbers indicate rank correlation coefficients (cc); numbers in parentheses indicate partial cc; */**/***: $p<0.05/p<0.01/p<0.001$, permutation test. **B-K**, Same as **A**, for other parameters. (**B**) DC gain is the mean firing rate during WN trials, divided by the mean spontaneous firing rate in the lack of light. (**C**) Fano factor is the variance/mean spike count over different WN trials. (**D**) Decoding quality, Q. (**E**) Filter width is the full width at half maximum (FWHM) of the filter. (**F**) Time lag. (**G**) Firing rate is the mean spontaneous firing rate. (**H**) ACH COM is the center of mass of the ACH. (**I**) Spike width is the trough-to-peak duration of the spike waveform. Firing rate, ACH COM, and spike width (**G-I**) are computed in the lack of light. (**J**) WN firing rate is the mean firing rate during WN trials. (**K**) Cell type is a binary indicator that equals 1 for DA PYR and 0 for IDA INT. (**L**) Summary of the cc's and partial cc's between precision and every tested parameter. Here and in **M,** error bars

indicate SEM. Filter width and time lag are positively correlated with precision, whereas decoding quality and WN firing rate are negatively correlated. Thus, precision is higher (lower values) when decoding quality is high (higher values), when the WN firing rate is high, when the filter is narrower, and when time lag is short. (**M**) The contribution of each parameter (the product of the rank regression coefficient and the cc; can be negative for some parameters) to the total variability of the precision explained by the model (R^2). Within the IDA INT population, the major source of contribution is from the WN firing rate. In contrast, the major source of contribution for DA PYR is filter width. For the combined population, the cell type parameter makes a minor contribution. Thus, WN firing rate is important in generating more precise IDA INT, but is not a major contributor for the difference in precision between the two populations.

(**N-U**) Error correction and improved precision are preserved when IDA INT firing rate during WN trials is equated to DA PYR rates. (**N**) CDFs of the baseline (BL) firing rate (the mean spontaneous firing rate, in the lack of light) of the DA PYR and IDA INT. Here and in **O-U**, vertical dashed lines show group medians; ns/***: p>0.05/<0.001, *U*-test. During baseline, IDA INT firing rates are an order of magnitude higher than DA PYR rates (medians: 6.9 vs. 0.6 spikes/s). (**O**) CDFs of the WN firing rates. Median IDA INT firing rates are approximately double the DA PYR firing rates (medians: 28 vs. 12 spikes/s; p<0.001, *U*-test). (**P**) For each IDA INT, WN firing rate was pruned by removing the spikes least likely to have emanated from the WN input. After pruning, the median firing rates of the IDA INT population are not higher than the firing rates of the DA PYR (DA PYR: 12 spikes/s; IDA INT: 11 spikes/s, pink dashed line). (**Q**) CDFs of the time lags of DA PYR and IDA INT after pruning. Akin to the IDA INT, pruned IDA INT exhibit longer time lags compared to the DA PYR (medians: 3.6 vs 2.4 ms; see **Figure 2F**). (**R**) Pruning does not change IDA INT decoding quality (medians: 0.19 vs. 0.19). (**S**) Distribution of the decoding quality between pairs of connected DA PYR and pruned IDA INT (see **Figure 2J**). The decoding quality of the pruned IDA INT is 0.098 higher than the decoding quality of the presynaptic DA PYRs (here and in **H**,***: p<0.001, Wilcoxon test), indicating error correction. (**T**) CDFs of the precision of the DA PYR and of the IDA INT after pruning. The pruned IDA INT exhibit high precision despite reduced firing rates. In particular, pruned IDA INT are more precise than DA PYR (median: 2.4 vs 3.2 ms; p<0.001, *U*-test; see **Figure 2H**). (**U**) Distribution of the precision difference between pairs of connected DA PYR and pruned IDA INT (see **Figure 2L**). The precision of the pruned IDA INT is 1.2 ms higher than the precision of the presynaptic DA PYRs.

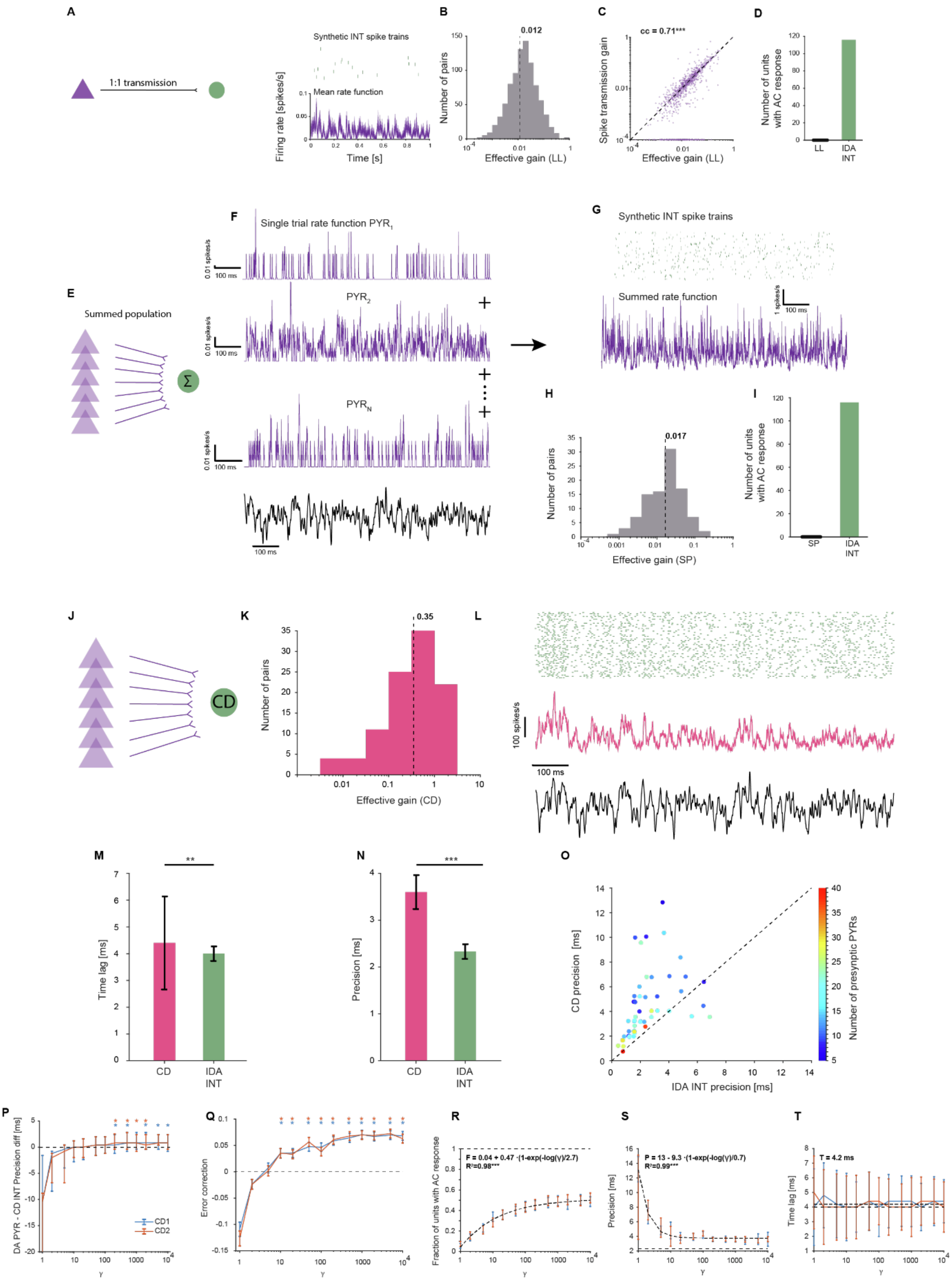

Figure S4. Coincidence detection transmission models produce error correction and temporally precise spiking in postsynaptic IDA INT. Related to Figure 4.

(**A-D**) Labelled line (LL) transmission model does not generate spiking in postsynaptic sINT. (**A**) *Left*, Illustration of a LL transmission model, showing a presynaptic neocortical DA PYR (purple triangle) and a postsynaptic IDA INT. *Right, bottom*, Mean sINT rate function, averaged over all trials (n=200) of the example DA PYR spike trains. *Right, top*, The resulting sINT spike trains during a subset of 50 WN trials. The sINT does not exhibit an AC response to the WN. (**B**) In the presence of a fixed input (the WN), the *effective* transmission

gain can be estimated by n_1/n_0, where n_0 (or n_1) refers to the number of presynaptic PYR spikes (or the number of sINT spikes produced, excluding background spikes) during WN. For the LL sINT spike trains, the effective gain is smaller than the spike transmission gain (median [IQR]: 0.012 [0.002 0.029]; p<0.001, Wilcoxon test; see **Figure 4C**). (**C**) Spike transmission gain and effective transmission gain are correlated (cc, 0.71; ***: p<0.001, permutation test). (**D**) Using the LL approach, none of the sINT exhibit an AC response.

(**E-I**) Summed population (SP) transmission model does not generate spiking in postsynaptic sINT. (**E**) Illustration of SP transmission model for an sINT (green), generated by linear summation of all inputs from the presynaptic DA PYR (purple triangles) to the modeled sINT. (**F**) For each presynaptic PYR, a rate function was generated. (**G**) For every trial, all rate functions were added together, producing a summed rate function (shown averaged over all trials; *bottom*). On every trial, the summed rate function was used to stochastically generate an sINT spike train. In the example, N=16 simultaneously-recorded presynaptic PYR were used. sINT spike trains during 50 trials are shown (*top*). (**H**) The effective gain between the summed presynaptic PYR and their postsynaptic sINT (median [IQR]: 0.017 [0.005 0.029], 109 pairs) is higher than the effective gain for the LL model (p=0.03, *U*-test). (**I**) Using the SP with added background activity, none of the sINT exhibit an AC response.

(**J-T**) coincidence detection (CD) transmission models generate precise spiking in postsynaptic sINT. (**J**) In the CD transmission models, inputs that arrive simultaneously are combined in a supra-linear manner. (**K**) The effective gain between the presynaptic PYR and their postsynaptic CD sINT (median [IQR]: 0.35 [0.10 0.78], 109 pairs) is higher than the effective gain for the SP model (p<0.001, Wilcoxon test). (**L**) Example of a CD generated rate function (*middle*, pink line) and the resulting sINT spike trains (*top*). (**M**) The time lag is longer for CD sINT, compared to IDA INT (medians: 4.4 vs. 4 ms; **: p<0.01, Wilcoxon test). (**N**) Precision of the CD sINT is poorer than that of the IDA INT (medians: 3.6 vs. 2.3 ms; ***: p<0.001, Wilcoxon test). (**O**) CD sINT and IDA INT precision are particularly similar when the number of simultaneously-recorded presynaptic PYR is large. (**P**) Two CD models were employed: CD1 (multiplicative nonlinearity) and CD2 (exponential summation). For both models, precision difference between pairs of connected DA PYR and the corresponding postsynaptic CD sINT was evaluated as a function of γ, a scalar non-negative non-linearity factor. The median difference is larger than zero (i.e., the CD model exhibits precision improvement) for $\gamma>20$. Precision improvement becomes consistent for $\gamma\geq200$ for both models (*: p<0.05, Wilcoxon test). Here and in **Q-T**, error bars indicate SEM over sINT. (**Q**) The median error correction is larger than zero for $\gamma>5$. Error correction becomes consistent for $\gamma\geq10$ for both models (*: p<0.001, Wilcoxon test). (**R**) For both models, the fraction of CD sINT that exhibit an AC response as a function of γ is monotonically increasing and achieves 95% of the asymptotic value (0.51) at $\gamma=127$ (51% of units exhibit an AC response; 95% confidence interval: [43% 0.51%]; fit to an exponential model: $R^2=0.99$, p<0.001, F-test). Here and in **S, T** the fit was for CD1. In **M-O**, CD1 was used with $\gamma=1000$. (**S**) The median CD sINT temporal precision improves as a function of γ, and reaches 95% of the asymptotic value (P=3.75 ms) at $\gamma=9$ (95% confidence interval, [3.56 3.94] ms; $R^2=0.98$, p<0.001). (**T**) The median CD sINT time lag is fixed, regardless of γ (constant fit to the data: T=4.2 ms).

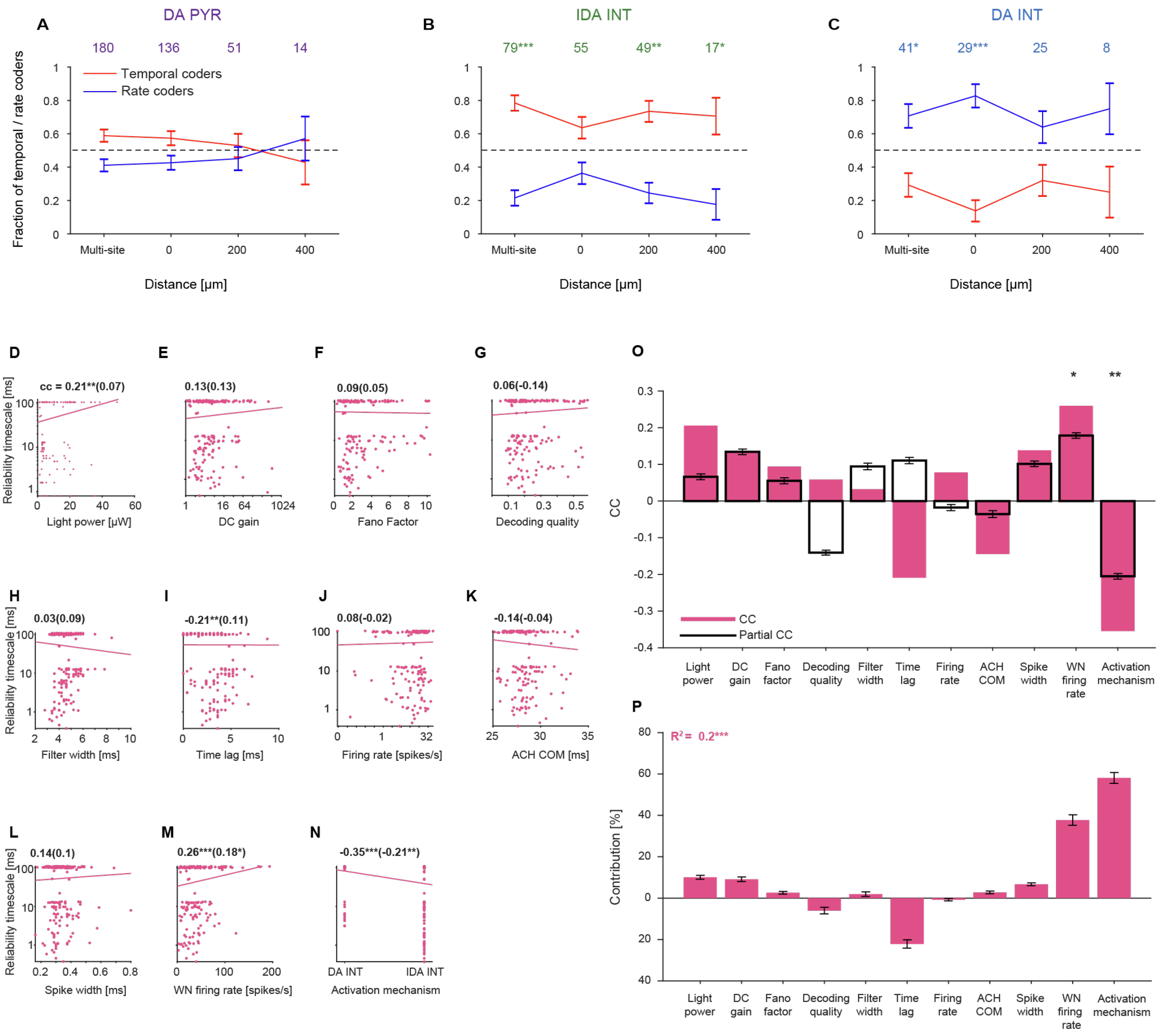

Figure S5. Reliability timescale of INT depends mainly on the activation mechanism. Related to Figure 5. (**A-C**) Reliability timescale of INT is independent of the distance from the illuminated shank. Fraction of rate (blue) and temporal coders (red) as a function of distance from the illuminated shank. Most IDA INT/DA INT are temporal/rate coders, respectively; DA PYR exhibit mixed coding behavior. */**/***: p<0.05/p<0.01/p<0.001, *G*-test.

(**D-P**) Reliability timescale of INT depends on WN firing rate and the activation mechanism. (**D**) Scatter plot of the reliability timescale and mean light power of the WN signal for a combined population of DA and IDA INT. (**E-M**), Same as **D**, for the same parameters used in **Figure S3B-J**. (**N**) Activation mechanism is a binary indicator that equals 0 for direct activation and 1 for indirect activation. (**O**) cc's and partial cc's between reliability timescale and every tested parameter. Here and in **P,** error bars indicate SEM. WN firing rate is positively correlated with reliability timescale, whereas activation mechanism is negatively correlated. Thus, reliability timescale is lower (tending towards temporal coding) when the WN firing rate is lower, and when the unit is indirectly activated. (**P**) The contribution of each parameter to the total variability of reliability timescale explained by the model (R^2). For the combined population, the activation mechanism makes the highest contribution.

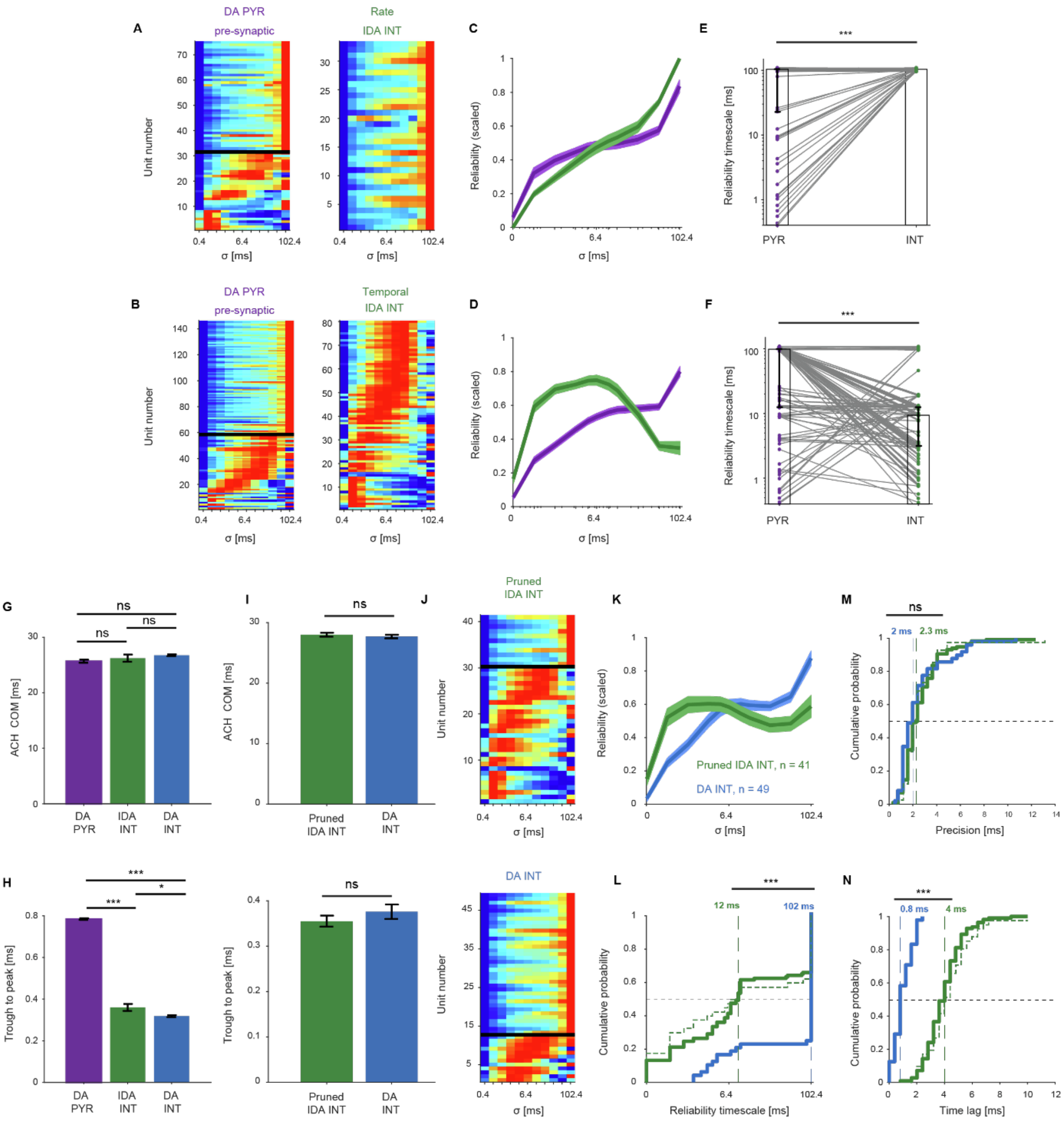

Figure S6. IDA INT reliability timescale is not inherited from DA PYR, and IDA INT are high precision temporal coders. Related to Figure 5.

(**A-F**) IDA INT reliability timescale is not inherited from DA PYR. (**A**) Stacked reliability profiles (scaled to the 0-1 range and sorted according to peak) of neocortical DA PYR (n=75; *left*) presynaptic to IDA INT classified as rate coders (n=33; *right*). Reliability profiles are arranged with temporal/rate coders below/above the black lines. (**B**) Same as **A**, for DA PYR (n=146; *left*) presynaptic to IDA INT classified as temporal coders (n=80; *right*). (**C**) The reliability profiles of the two populations, averaged (mean±SEM) over all "rate IDA INT" and their presynaptic DA PYR. At the group level, DA PYR presynaptic to "rate IDA INT" act as rate coders. (**D**) Same as **C**, for temporal IDA INT. At the group level, the DA PYR presynaptic to "temporal IDA INT" also act as rate coders. (**E**) The reliability timescale of each rate IDA INT is plotted, along with the reliability timescale of all corresponding presynaptic PYRs. Grey lines indicate inferred monosynaptic connectivity. In 54/75 (72%) of the pairs, the reliability timescales are the same. In all other cases, the reliability timescale of the presynaptic PYRs is lower than that of their IDA INT peers. Thus, the null hypothesis of similar reliability timescales for the pre- and postsynaptic units is rejected for this population (***: $p<0.001$, Wilcoxon test). (**F**) Same as **E**, for temporal IDA INT. In 113/146 (77%) of the pairs, the reliability timescale is lower for the IDA INT than for the presynaptic DA PYR (***: $p<0.001$, Wilcoxon test).

(**G-N**) Neocortical IDA INT with timing and waveform properties of optically-verified PV cells are high precision temporal coders. (**G-H**) DA INT and IDA INT have similar second-order spontaneous spike timing statistics (quantified here using the ACH center of mass, *left*) but may differ in their spike waveforms (quantified

using the trough-to-peak duration, *right*). IDA INT and DA INT are distinct from DA PYR. ns/*/***: p>0.05/p<0.05/0.001, *U*-test. (**I**) The neocortical IDA INT population (n=116) was pruned such that the remaining IDA INT ("pruned IDA INT"; n=41) and all DA INT (n=49) had similar spike timing (*top*) and spike waveforms (*bottom*). (**J**) Stacked reliability profiles of pruned IDA INT (*top*) and DA INT (*bottom*) populations, scaled and sorted according to the timescale of the peak. (**K**) Same as **C** for the two population. At the group level, DA INT act as rate coders, whereas pruned IDA INT act as temporal coders. (**L**) CDFs of the global maxima of the reliability profiles of the two populations. Here and in **M** and **N**, vertical dashed lines show group medians; ns/***: p>0.05/p<0.001, *U*-test. The pruned IDA INT exhibit reliability timescales consistent with temporal coding, as does the complete population of IDA INT (green dashed line). (**M**) DA INT and pruned IDA INT exhibit similar precision. (**N**) CDFs of the time lags of the two populations. The median time lag for DA INT is 3.2 ms shorter than for pruned IDA INT.

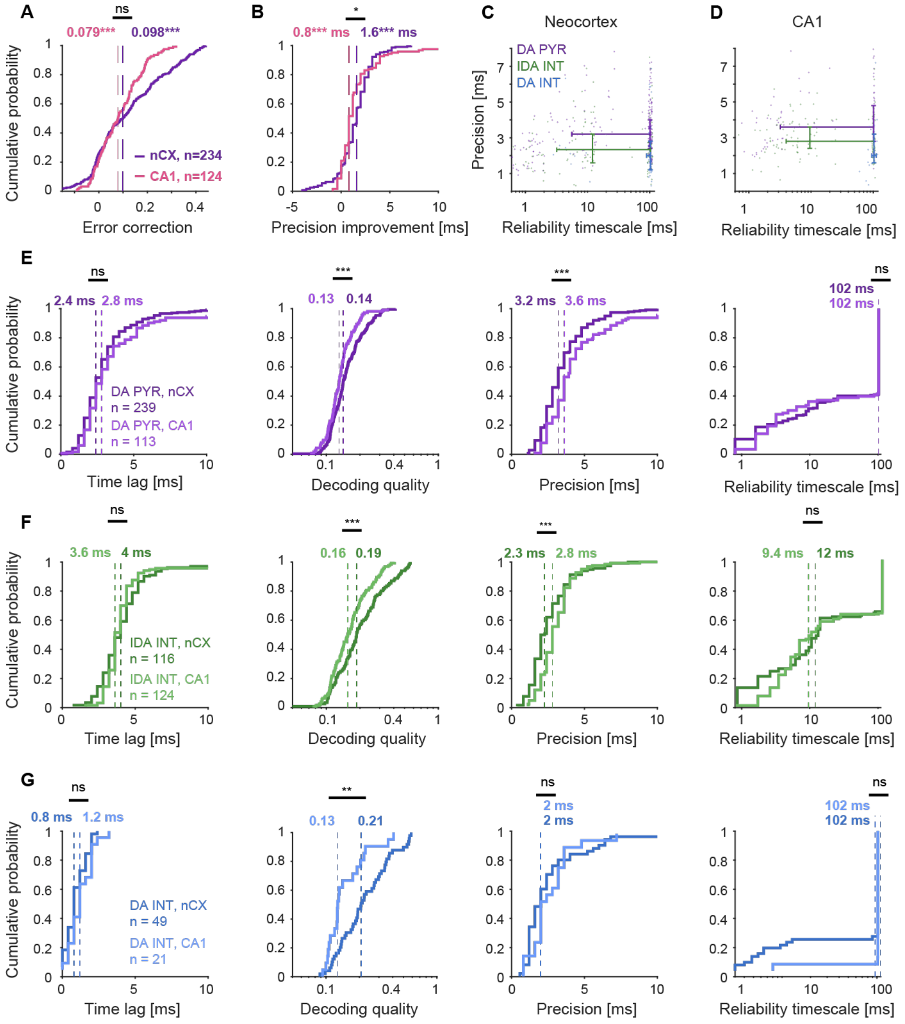

Figure S7. Precision improvement is larger in neocortex than in CA1. Related to Figure 7.

(**A**) Error correction between pairs of connected DA PYR and IDA INT is evident in neocortex (purple) and in CA1 (pink). Error correction is not consistently different between neocortex and CA1. Here and in **E**, ***: p<0.001, Wilcoxon test; ns/*: p>0.05/p<0.05, *U*-test.

(**B**) Precision improvement between connected DA PYR and IDA INT is higher in the neocortex than in CA1.

(**C-D**) Summary of the relations between precision and reliability timescale for all neocortical and CA1 units. Error bars represent population medians and IQR. DA PYR resemble low precision rate coders (cf. **Figure 1G**); IDA INT resemble high precision temporal coders (**Figure 1H**); and DA INT resemble high precision rate coders (**Figure 1I**). In both regions, INT exhibit higher precision than PYR, and DA INT act as rate coders. IDA INT act as temporal coders and exhibit higher precision than DA PYR.

(**E**) Neocortical DA PYR exhibit higher decoding quality and precision than DA PYR in CA1 (*center*) while having similar time lags (*left*) and reliability timescales (*right*). Here and in **F-G**, ns/**/***: p>0.05/p<0.01/p<0.001, *U*-test).

(**F**) Neocortical IDA INT exhibit higher decoding quality and precision than IDA INT in CA1, while exhibiting similar time lags and reliability timescales.

(**G**) Neocortical DA INT exhibit higher decoding quality than DA INT in CA1, while having similar time lags, precision and reliability timescales.

Animal ID	Opsins	Probe	Light sources	Sessions	PYR	INT	AC response PYR	AC response INT
**Neocortex**								
mC41	CaMKII::ChR2	Stark64	5x470 nm, 1x356 nm LEDs	5	119	21	42	8
mF84	CaMKII::ChR2	Linear32	1x470 nm LED	5	48	19	4	4
mF93	CaMKII::ChR2	Stark64	5x470 nm LEDs	3	161	87	26	9
mF108	CaMKII::ChR2	Stark64	4x470 nm LEDs	2	75	62	31	26
mA234	CaMKII::ChR2	Buzsaki32	3x470 nm LEDs	11	359	132	62	21
mDS1	CaMKII::ChR2	Dual-sided64	2x470 nm LEDs	5	46	31	26	5
mDS2	CaMKII::ChR2	Dual-sided64	2x470 nm LEDs	8	1059	211	358	91
mDL5	PV::ChR2	Buzsaki32	2x470 nm LEDs, 1x450 nm LDs	14	159	62	0	23
mP77	PV::ChR2	Buzsaki32	2x470 nm LEDs	5	26	50	0	7
mP151	PV::ChR2	Buzsaki32	2x470 nm LEDs	7	44	25	0	4
mP20	PV::ChR2	Stark64	6x470 nm LEDs	4	97	40	0	5
mP31	PV::ChR2	Stark64	4x470 nm LEDs	8	432	103	0	27
**CA1**								
mC41	CaMKII::ChR2	Stark64	5x470 nm, 1x356 nm LEDs	21	1535	265	345	65
mDS1	CaMKII::ChR2	Dual-sided64	2x470 nm LEDs	10	397	33	106	11
mDS2	CaMKII::ChR2	Dual-sided64	2x470 nm LEDs	3	126	57	78	19
mA234	CaMKII::ChR2	Buzsaki32	3x470 nm LEDs	21	1299	180	126	58
mP23	PV::ChR2	Buzsaki32	2x470 nm LEDs, 2x450 nm LDs	2	36	8	0	3
mP101	PV::ChR2	Buzsaki32	4x470 nm LEDs	6	159	29	0	7
mP151	PV::ChR2	Buzsaki32	2x470 nm LEDs	12	27	30	0	11
mP30	PV::ChR2	Stark64	4x470 nm LEDs	3	132	30	0	0
mP31	PV::ChR2	Stark64	4x470 nm LEDs	7	280	85	0	9

Table S1. Unit yield from every experimental animal. Related to STAR Methods.
